# A Seven Amino Acid Long Region in the Cardiac Ca_v_1.2, Encompassing Ser1487, Is Essential for Mice Embryonic Development and “Fight or Flight” Responses

**DOI:** 10.3390/cells15181655

**Published:** 2026-09-14

**Authors:** Catherine Jenkins, Henrietta Cserne Szappanos, Agnieszka Dyrda, Teagan S. Er, Filip van Petegem, Livia C. Hool

**Affiliations:** 1 Ben Beale Laboratory in Cardiovascular Research, School of Human Sciences, The University of Western Australia, M309, 35 Stirling Highway Perth, WA 6009, Australia; cathy.jenkins@uwa.edu.au (C.J.); henrietta.csernedrszappanos@qimrb.edu.au (H.C.S.); agnieszka.dyrda@uwa.edu.au (A.D.); teagan.er@research.uwa.edu.au (T.S.E.); 2 Department of Biochemistry and Molecular Biology, Life Sciences Institute, University of British Columbia, 2350 Health Sciences Mall, Vancouver, BC V6T 1Z3, Canada; filip.vanpetegem@ubc.ca; 3 Victor Chang Cardiac Research Institute, 405 Liverpool Street, Darlinghurst, NSW 2010, Australia

**Keywords:** Fight or Flight, Cardiac Ca_v_1.2, β-adrenergic receptor signaling, PKA phosphorylation

## Abstract

**Highlights:**

**What are the main findings?**

- A 7aa region in the proximal C-terminus is essential for embryonic development and the structural integrity of Ca_v_1.2.
- Mutation of S1487 to alanine attenuates the increase in I_CaL_ following β-adrenergic receptor stimulation in vitro and in vivo.

**What are the implications of the main findings?**

- We have identified a critical region required for normal channel integrity and cardiac development.
- We have demonstrated that phosphorylation of S1487 by PKA within the 7aa region is essential for “Fight or Flight” responses, clarifying the molecular mechanism.

**Abstract:**

The cardiac calcium channel Ca_v_1.2 is essential for embryonic development, cardiac excitation–contraction coupling, and the “Fight or Flight” response. Regulation of Ca_v_1.2 by protein kinase A (PKA) phosphorylation is critical to this process, though the underlying mechanism remains contentious. We previously identified serine 1458 (S1458) in the proximal C-terminus of the human Ca_v_1.2 as essential for PKA-mediated regulation in vitro. To investigate its functional role in vivo, we generated three mouse models targeting S1487, the mouse equivalent of human S1458. Comprehensive phenotyping and assessment of responses to β-adrenergic receptor stimulation were conducted in vivo, ex vivo, and in vitro. We demonstrate for the first time that a seven amino acid (7aa) region containing S1487 is crucial for proper channel folding, as homozygous mutant mice exhibited embryonic lethality. We confirm that phosphorylation at S1487 is necessary for altered Ca_v_1.2 function required for the “Fight or Flight” response, clarifying the functional significance of this region.

## 1. Introduction

The cardiac L-type calcium channel is essential for life, and mice homozygous null for the Ca_v_1.2 gene are embryonic lethal [1]. The Ca_v_1.2 protein (α1C subunit) forms the channel pore, the primary route for calcium influx into cardiac myocytes, initiating contraction and regulating excitation. It also plays a major role in β-adrenergic responses, including the “Fight or Flight” response, an important physiological response to stress. Its carboxyl terminus contains binding sites for calmodulin, junctophilin, and A kinase anchoring proteins (AKAPs) [2,3,4], which regulate properties including calcium-dependent inactivation and functional responses to phosphorylation by protein kinases. It has been demonstrated that some serine residues, including serine 1700, 1704, and 1928, are not necessary for β-adrenergic responses in mice but may be necessary for channel regulation [5,6,7,8]. The long NT isoform of Ca_v_1.2 contains 158 serine and 112 threonine residues in total; however, their functional relevance following β-adrenergic receptor stimulation remains contentious. Recently, using a proximity ligand assay, an integral player in the “Fight or Flight” response was proposed, a small GSK molecule known as Rad [9]. However, the mouse model used to demonstrate the importance of this molecule in β-adrenergic responses was complex and included introduction of rabbit and human DNA, simultaneous mutations in 35 amino acids in the rabbit α1C subunit of Ca_v_1.2, and doxycycline-induced expression of protein engineered in a dihydropyridine (DHP)-insensitive channel.

In contrast, we used a much-simplified approach to identify the critical serine. We studied the purified human Ca_v_1.2 channel reconstituted into artificial liposomes [10]. The major benefit of this model is that channel function is studied in isolation from the auxiliary subunits and regulatory pathways [11]. Using this approach, we found that none of the previously postulated serine residues in the distal C-terminal domain are necessary for PKA phosphorylation and increase in calcium current and that mutating serine residues in the Repeat I-II and II-III linker regions did not alter the effect of PKA phosphorylation. However, an alanine substitution at serine 1458 (S1458A) in the proximal carboxyl region prevented phosphorylation, assessed using semi-quantitative fluorescent phosphoprotein detection and MS/MS mass spectrometry analysis of the residue, and attenuated the increase in single channel mean open probability of Ca_v_1.2 channels. S1458, in the truncated C-terminal short-NT isoform of the human cardiac Ca_v_1.2 channel (corresponding to S1535 in Q13936-1), is homologous to S1517 in murine neurons [12] and S1570 in rabbit heart [13], which also mediate calcium current following exposure to PKA.

This finding prompted us to create mouse models to examine the functional significance of the serine in vivo. Using CRISPR-Cas9 technology, we generated three mouse models targeting S1487, the mouse homologue of the human S1458. We first replicated the alanine substitution, S1487A, which prevents PKA phosphorylation at this site. We also created a phosphomimetic mutant with glutamic acid substitution at site 1487 (S1487E) that mimics constitutive phosphorylation at this site. Coincidentally, a small deletion of a 21 base-pair region occurred spontaneously, affecting seven amino acids (7aa del), including S1487. We undertook extensive phenotyping of the mice in vivo and ex vivo. We found that this 7aa region is critical for the correct function and folding of the channel because mice homozygous for the 7aa deletion were embryonic lethal. In addition, our data confirm our previous in vitro studies, which found that S1487 is essential for PKA phosphorylation and altered Ca_v_1.2 function in response to β-adrenergic receptor stimulation.

## 2. Materials and Methods

### 2.1. Ethics

All animal work in this study was approved by The Animal Ethics Committee of The University of Western Australia in accordance with the Australian Code of Practice for the Care and Use of Animals for Scientific Purposes (NHMRC, 9th Edition, 2018).

### 2.2. Mouse Model

All mutant mouse models were generated using CRISPR-Cas 9 gene editing at the Walter and Eliza Hall Institute of Medical Research, Parkville, Victoria, Australia. Four intronic sgRNAs were used, including two sgRNAs upstream and two downstream of the targeted exon. In addition, the PAM sites of these sgRNAs (present in non-conserved genomic regions) were mutated to ensure that the targeted allele did not get cleaved by Cas9. The deletion of 21bp ACTAGGGATTGGTCTATCCTC (c119148258-119148238 Mus musculus strain C57BL/6J chromosome 6, GRCm39) occurred spontaneously. The corresponding amino acids deleted in this region were threonine, arginine, aspartic acid, tryptophan, serine, isoleucine, and leucine. Genotype-negative mice are referred to as wildtype (wt) mice.

### 2.3. In Vivo Phenotyping of Ca_v_1.2 Mutations

Male and female mice carrying the S1487A^+/+^, S1487E^+/−^, or 21bp deletion (7aa del)^+/−^ mutations in the CACNA1C gene were phenotyped up to 52 weeks for baseline parameters. Parameters included breeding and survival rates and echocardiography and electrocardiogram assessment of hearts of S1487A^+/+^, S1487E^+/−^, and 7aa del^+/−^ mutant mice. Heart weight/tibia length measurements were also recorded in adult mice and fetuses that died perinatally. Responses to β-adrenergic receptor stimulation were investigated in adult (20–52-week-old) S1487A^+/+^, S1487E^+/−^, and 7aa del^+/−^ mice following treatment with acute (20 mg/kg intraperitoneal injection once) or chronic (20 mg/kg intraperitoneal injection daily for two weeks) isoproterenol hydrochloride (ISO) (I6504, Sigma Aldrich, St. Louis, MO, USA).

### 2.4. Echocardiography and Electrocardiography Studies

M-Mode echocardiographic studies were performed on mice under light methoxyflurane anesthesia with the use of an i13L probe in the long axis on a VividTM 7 IQ ultrasound system (GE Healthcare, Buckinghamshire, UK), as previously described [14]. Each *n* represents the average of 3 quantitative measurements from each mouse for each treatment group. Measurements of interventricular septum thickness in diastole (IVSd), interventricular septum thickness in systole (IVSs), left-ventricular internal diameter in diastole (LVIDd), left-ventricular internal diameter in systole (LVIDs), left-ventricular posterior wall thickness in diastole (LVPWd), left-ventricular posterior wall thickness in systole (LVPWs), ejection fraction (EF%), and fractional shortening (FS%) were all conducted at a sweep speed of 200 mms^−1^. Fractional shortening was calculated using the formula [(LVIDd − LVIDs)/EDD] × 100.

Electrocardiograms (ECGs) were collected from anaesthetized mice placed on a heating pad. ECGs were recorded using an Animal BioAmp system and PowerLab software (v8.1.13) using subdermal needle electrodes at the lead II position. Baseline measurements were recorded for 10 min prior to treatment with isoproterenol. ECG parameters were assessed from 10 s of contiguous data, and the corrected QT interval (QTc) was obtained using the Mitchell formula [15].

### 2.5. Western Blots

Total heart homogenate was pooled from groups of five mice of each sex. Briefly, snap frozen hearts were weighed, ground into a fine powder in liquid N_2_ using a mortar and pestle, and homogenized in 1:4 RIPA buffer consisting of (in mM) NaCl 150, Tris 50, Na_4_P_2_O_7_ 20, Na_3_CO_4_ 2, NaF 1, 0.5% Na deoxycholate, 1% Triton X-100, 0.1% SDS, pH 7.4, supplemented with cOmplete™, Mini, EDTA-free Protease (Roche, Sydney, Australia, 4693159001), and PhosStopTM phosphatase (Roche, 4906837001, 1:100 dilution) inhibitor tablets. Total heart homogenate was then centrifuged at 10,000× *g* for 5 min at 4 °C. The protein concentration of all samples was quantified using the Bradford protein assay using BSA as a standard. Samples (25 µg) were loaded into precast 10% Bio-RAD Mini-PROTEAN^®^ TGX Stain-Free^TM^ SDS-polyacrylamide gel (Bio-RAD Laboratories, Sydney, Australia) and then electrophoretically transferred to 0.2 µm nitrocellulose membrane (Trans-Blot^®^ TurboTM Transfer Pack, Bio-RAD Laboratories, Sydney, Australia) using the Bio-RAD Trans-Blot^®^ TurboTM Transfer System. Blots were stripped between each re-probing step using stripping buffer, consisting of (in mM) 62.5 Tris HCl (pH 6.8), 100 2-mercaptoethanol, and 2% SDS for 30 min at 50 °C. Quantitative densitometry was performed on images captured using a Chemidoc imaging system (Bio-RAD, Sydney, Australia) and ImageJ software. Data are presented as optical density of protein expression, normalized to loading control detected on the same blot. Blots were carried out in triplicate. Full-length gels for all Western blots are presented in Supplementary Figures S2 and S3. Refer to Table 1 for full list of antibodies used.

### 2.6. Isolation of Adult Mouse Cardiac Myocytes

Myocytes were isolated from 15–30-week-old male and female S1487A, S1487E, 7aa del, and wildtype mice as previously described [16,17]. Mice were first anaesthetized with methoxyflurane before administration of an intraperitoneal injection of pentobarbitone sodium (240 mg/kg). Hearts were rapidly excised, cannulated via the aorta, and mounted on the Langendorff apparatus. Hearts were retrogradely perfused with Krebs–Henseleit buffer (KHB) containing (in mM) 120 NaCl, 25 NaHCO_3_, 4.8 KCl, 2.2 MgSO_4_, 1.2 NaH_2_PO_4_, and 11 glucose (pH 7.35 with O_2_/CO_2_ at 37 °C) for 4 min at 37 °C. Digestive enzyme collagenase B (2.4 mg/mL) was added to the KHB, and hearts were perfused for 3 min before the addition of 40 µM CaCl_2_. Hearts were then perfused for a further 8 min in the presence of collagenase and calcium. The aorta, atria, and right ventricle were removed, and the left ventricle was gently teased apart and triturated to dissociate myocytes suspended in KHB. The cell suspension was allowed to settle for 20 min before the supernatant was removed. Myocytes were resuspended in calcium-free HEPES-buffered solution (HBS) containing (in mM) 5.3 KCl, 0.4 MgSO_4_·7H_2_O, 139 NaCl, 5.6 Na_2_HPO_4_·2H_2_O, 5 glucose, 20 HEPES, and 2-glutamine (pH 7.4 at 37 °C). Calcium was titrated into the cell suspension to achieve a final concentration of 1 mM. An aliquot of 1 mL of cell suspension was further titrated to a calcium concentration of 2.5 mM, which was then used for patch clamp studies.

### 2.7. Patch Clamp Studies in Cardiomyocytes

L-type calcium channel currents (I_CaL_) in intact ventricular cardiomyocytes were measured using whole-cell configuration of the patch clamp technique. Currents were measured in extracellular modified Tyrode’s solution containing (in mM) 140 NaCl, 5.4 CsCl, 1 CaCl_2_, 0.5 MgCl_2_, 5.5 HEPES, and 11 glucose (pH adjusted to 7.6 with NaOH at room temperature). All recordings were performed at 37 °C. Microelectrodes with tip diameters 3–5 µm and resistances of 2–4 MΩ contained the following (in mM): 115 CsCl, 10 HEPES, 10 EGTA, 20 tetraethylammonium chloride, 5 MgATP, 0.1 Tris-GTP, 10 phosphocreatine, and 1 CaCl_2_ (pH adjusted to 7.05 at 37 °C with CsOH). Macroscopic currents were recorded using an Axopatch 200B voltage-clamp amplifier (Molecular Devices, San Jose, CA, USA) and a Digidata 1440A interface with pClamp9 software (v11.2.2) (Molecular Devices, San Jose, CA, USA). An Ag/AgCl electrode was used to ground the bath. Once whole-cell configuration was achieved, the holding potential was set at −50 mV. Sodium and T-type calcium channels were inactivated by applying a 50 ms pre-pulse to −30 mV immediately before each test pulse. To test the β-adrenergic response, 100 nM ISO was added to the bath solution during stable recording. Current/voltage (I/V) recordings were taken before and after ISO using step membrane depolarization from −50 mV to 60 mV in 10 mV increments.

### 2.8. Calcium Transients in Cardiomyocytes

Isolated cardiomyocytes were incubated with 5 μM Fluo-4 AM in HBS solution containing 1 mM Ca^2+^ (Life Technologies, Carlsbad, CA, USA, F14217) for 20 min at 37 °C. Following incubation, the solution was carefully removed and replaced with HBS containing 1 mM Ca^2+^ without Fluo-4. Fluo-4 fluorescence was then recorded in HBS at 37 °C in stream acquisition mode, recording 400 images at 10 ms intervals using a Zyla 5.5 sCMOS camera attached to an inverted Nikon TE2000-U microscope (excitation 480 nm, emission 535 nm). The signal was quantified by manually tracing cardiomyocytes using MetaMorph^®^ software version 7.10.3. An equivalent region not containing cells was used as the background and subtracted. The signal for each cell (F) was normalized to the basal signal (F_0_), yielding F/F_0_; therefore, the basal signal approximated 1.0.

### 2.9. HEK293 Cell Lines

Flp-In™ 293 T-REx cell lines (Thermo Fisher Scientific, Brisbane, Australia, R78007) stably expressing CACNB2b, CACNA2D1, and CACNA1C-wt or CACNA1C-S1458A were prepared as previously described [18]. The cell lines were kept at 37 °C in a humidified atmosphere with 5% CO_2_, continuously maintained and passaged in sterile culture flasks containing DMEM/F12 (Thermo Fisher Scientific, Brisbane, Australia, 11995065) medium supplemented with 10% fetal bovine serum (Fisher Biotec, Perth, Australia, FBS-AU-015), 1.0% penicillin/streptomycin (Thermo Fisher Scientific, Brisbane, Australia, 1037801), 100 μg/mL of hygromycin B (Thermo Fisher Scientific, Brisbane, Australia, 10687010), 10 μg/mL of blasticidin (Sigma, Melbourne, Australia, 262072) and 0.25 μg/mL of puromycin (Sapphire Bioscience, Sydney, Australia, S7417). Channel expression was induced using 0.2 mg/mL of doxycycline (Sigma, Melbourne, Ausralia, D9891) at least 18 h before the experiment.

### 2.10. Transient Transfection in HEK293 Cell Line

Flp-In™ 293 T-REx (Thermo Fisher Scientific, Brisbane, Australia, R78007) cell lines stably expressing CACNB2b, CACNA2D1, and CACNA1C-wt or CACNA1C-S1458A (a kind gift from Dr Chai-Ann Ng, Victor Chang Cardiac Research Institute) were transiently transfected with 1 μg of plasmid encoding mouse Rad protein using Lipofectamine 3000 (Thermo Fisher Scientific, Brisbane, Australia, L3000008), according to the manufacturer’s instructions, 24 h before induction of channel expression.

### 2.11. Plasmids

Two Rad plasmids were used in this study: the commercially available CFP_Rad_pcDNA4_HisMaxC (Addgene #41654) and Rad_pcDNA4_HisMaxC. The latter was generated from CFP_Rad_pcDNA4_HisMaxC by amplifying the plasmid without the CFP sequence using primers flanking the KpnI-BamHI region. The forward primer (linking to KpnI-BamHI) was 5′-CAGTGGGCGGTG-3′. The reverse primer (linking to KpnI-BamHI) was 5′-GATCCACCGCCCACTGGTAC-3′. Two separate PCR products were generated and then used as templates for overlapping PCR reactions. The integrity of the Rad plasmids was confirmed through Sanger sequencing (Eurofins Genomics, Louisville, KY, USA).

### 2.12. Patch Clamp Studies in HEK293 Cell Line

Ba^2+^ currents (I_Ba_) were recorded at room temperature using the whole-cell configuration of the patch clamp technique. The bath solution contained (in mM) NaCl 100, KCl 4, NMDG 40, BaCl_2_ 5, MgCl_2_ 1, HEPES 10, glucose 5, and sorbitol 5 with pH 7.40 (HCl). The patch electrode had resistances between 4 and 5 MΩ when filled with the pipette solution consisting of (in mM) 108 Cs methansulfonate, 4.5 MgCl_2_, 5 Na_2_-phosphocreatine, 5 creatine, 5 pyruvate, 5 oxalacetate, 10 Na_2_ATP, 24 HEPES, and 10 EGTA, with pH 7.25 (CsOH). Cells were kept at −80 mV holding potential, and 150 ms depolarizing step potentials were applied with Axon™pCLAMP™ 10.7 Electrophysiology Data Acquisition & Analysis Software (Molecular Devices) using an Axopatch 200B Amplifier (Axon; Molecular Devices, San Jose, CA, USA), low-pass filtered at 5 kHz and digitized with the Digidata 1322 A (with 25 kHz sampling frequency). Access resistance was kept < 15 MΩ, and leak subtraction was performed online (P/4 protocol). To reveal the β-adrenergic effect, the I_Ba_ were recorded at one depolarization step to 10 mV every 15 s, and 5 μM forskolin (Frsk)(Sigma, Melbourne, Australia, F6886) was applied to the external solution once the basal current had stabilized. The I/V curves were obtained before and after Frsk addition, at stable I_Ba_, through step membrane depolarization from −50 mV to 60 mV in 10 mV increments.

### 2.13. Data Analysis

All in vivo and in vitro data were first separated by sex to identify any effects of sex dimorphism in this study. No significant differences were observed; thus, male and female data were pooled for the duration of the study. Clampfit (Molecular Devices) version 11.2 was used for analysis of patch clamp recordings. The kinetic properties of activation and inactivation of Ca^2+^ current (in cardiomyocytes) and Ba^2+^ current (in HEK cells) were determined by fitting the rising phase and falling phase of the maximum sweep with a single-exponential function:

$$I=A\times e^{-\frac{t}{\tau }}+C,$$

where *I* is the current, *A* is the individual current amplitudes, and *τ* is the specific time constants of the current amplitudes.

GraphPad Prism (10.4.1) was used to fit the current voltage dependence according to

$$I\left(V\right)=\frac{G_{max}\times (V-V_{rev})}{e^{-\frac{V-V_{1/2}}{k}}+1},$$

where *G_max_* is the maximum conductance of the Ca_v_1.2, *V* is the imposed membrane potential, *V_rev_* is the extrapolated reversal potential of the current, *V*_1/2_ is the potential for half-maximal conductance, and *k* is the slope.

The conductance (*G*) was extrapolated using

$$G(V)=-\frac{I\times 1000}{V_{rev}-V}.$$

The voltage dependence of the barium (Ba^2+^) or calcium (Ca^2+^) conductance was fitted according to a Boltzmann distribution:

$$G(V)=\frac{G_{max}}{e^{-\frac{V-V_{1/2}}{k}}+1}$$

### 2.14. Statistical Analysis

All data are reported as mean ± standard error of the mean (SEM). Data were first tested for normality using the Shapiro–Wilk test, and equality of variances was then tested using the Browne–Forsythe test. Comparisons were made using Welch’s ANOVA for normally distributed data with Dunnett’s post hoc test for multiple comparisons to compare groups to a control. The Kruskal–Wallis test with Dunn’s post hoc test was carried out for comparisons between groups. Statistical significance was accepted at *p* < 0.05. Statistical analyses were carried out using GraphPad Prism software, version 10.4.1. (GraphPad Software, Boston, MA, USA).

## 3. Results

### 3.1. The 7aa Region of the Proximal C-Terminal Domain of the Ca_v_1.2 in Mice Is Critical for Embryonic Development and Survival

In vivo phenotyping was carried out in three mutant models and compared to wt. These included the phospho-null alanine substitution at S1487 (S1487A), the phosphomimetic glutamic acid substitution (S1487E), and the spontaneously occurring deletion of a seven amino acid sequence (TRDWSIL) (7aa del) surrounding S1487. Both S1487A and S1487E models enabled elucidation of the role of this critical serine in channel regulation and function. Additionally, our 7aa del model provided a unique opportunity to investigate the function of this region, as this sequence is highly conserved among species, including humans (Supplementary Figure S1), and the structure of this region has not been fully resolved.

**Figure 1:**
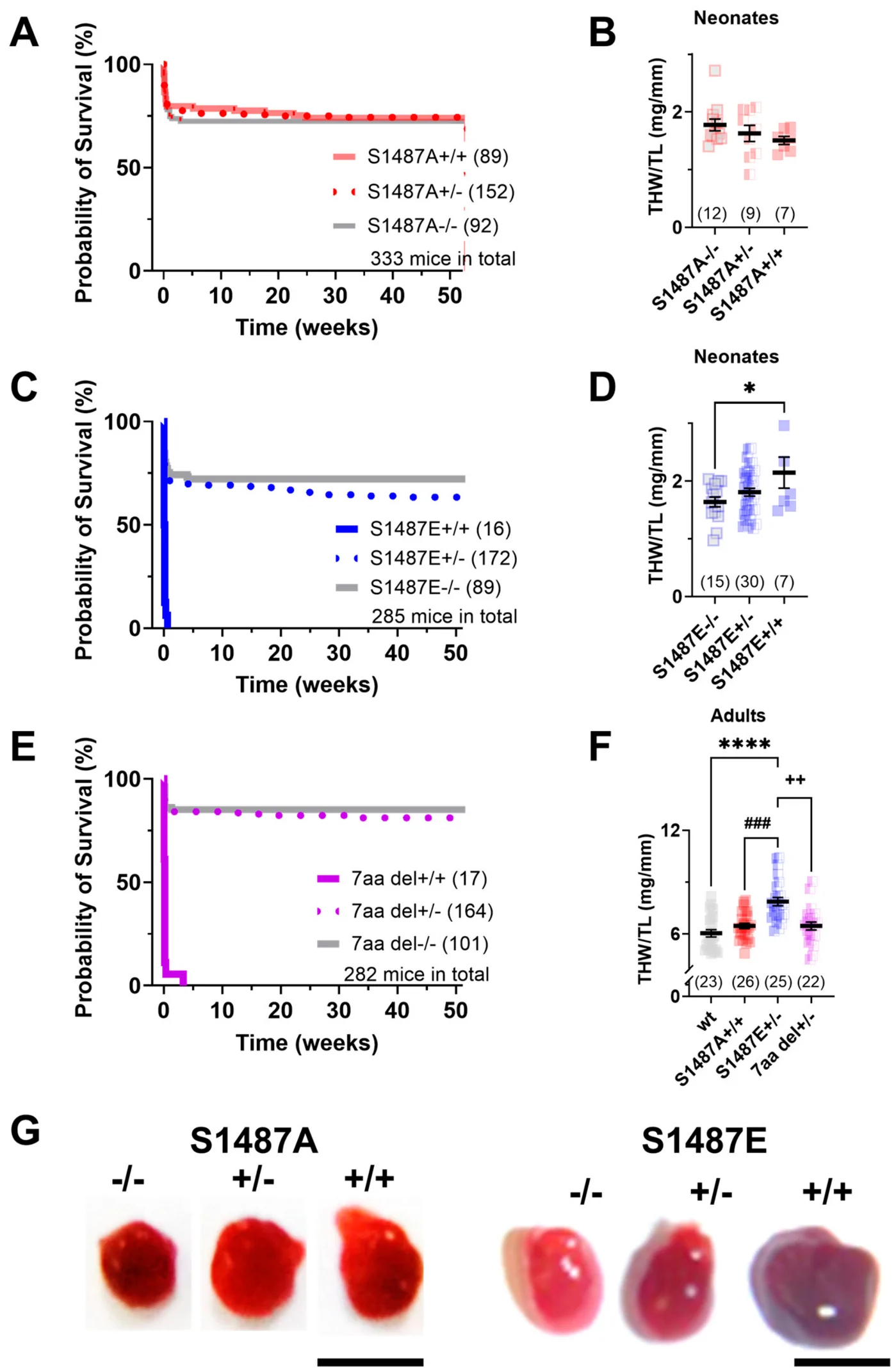
**Probability of survival and THW/TL measurements in S1487A, S1487E, and 7aa del mice.** Expression of the S1487A mutation resulted in viable pups (**A**) and no changes in THW/TL (**B**) in neonatal mice. Mice homozygote for the S1487E mutation were embryonic lethal (**C**) and developed an increased THW/TL ratio (**D**). The homozygote 7aa del mutation was also embryonic lethal (**E**). Analysis of THW/TL ratio in 52-week-old surviving mice showed increased THW/TL ratio in S1487E^+/−^ mice compared to all other genotypes (**F**). THW/TL comparisons in neonates were performed using one-way ANOVA and in adults using Welch’s ANOVA with Dunnet’s post hoc test for multiple comparisons. The number of samples in each group is labelled within the graphs. (**G**) Representative neonatal hearts excised from the genotypes as shown. Scale bar = 4 mm. (* = vs wt, # = vs S1487A^+/+^, and + = vs 7aa del^+/−^). * *p* < 0.05, **** *p* < 0.0001 (THW/TL: total heart weight to tibia length ratio). Symbols denote group comparisons and number of symbols denotes level of significance.

Results of survival analysis and in vivo phenotyping revealed that mice homozygous for an alanine substitution at S1487 (S1487A^+/+^) were viable at 52 weeks. However, mice with homozygous glutamic acid substitution, S1487E^+/+^ were embryonic lethal (Figure 1A,C). Post mortem analysis of embryos that carried the S1487E^+/+^ mutation and died perinatally revealed that their hearts were hypertrophic, demonstrated as increased total heart weight to tibia length ratio (THW/TL), compared to wt controls (Figure 1D). Heterozygous S1487E^+/−^ mice were viable at 52 weeks but also developed hypertrophy compared to wt controls (Figure 1F). Homozygous deletion of 21 base pairs (7aa del^+/+^) was not compatible with life (Figure 1E), and we could not obtain data on the effect of the 7aa del^+/+^ mutation on the THW/TL ratio perinatally, as embryos were lost very early in development.

We utilized the AlphaMissense platform to predict the pathogenicity of the S1487 mutations (corresponding to S1535 in Q13936-1 isoform) [19,20]. Consistent with our in vivo data, the S1535E and 7aa del mutations were predicted to be “likely pathogenic”. A pathogenicity score of “ambiguous” was assigned to S1535A, which was supported by our results demonstrating that this mutation is not lethal. Overall, the survival data emphasize the functional importance of the region surrounding S1487. The resulting impact on Ca_v_1.2 function is so detrimental that homozygosity of the S1535E and 7aa del mutations leads to embryonic lethality. Hypertrophy was observed in S1487E^+/+^ neonates and S1487E^+/−^ adult mice, confirming an adverse cardiac phenotype.

### 3.2. The 7aa Region Confers Stability in the Linker Between IVS6 and the EF-Hand Domain

**Figure 2:**
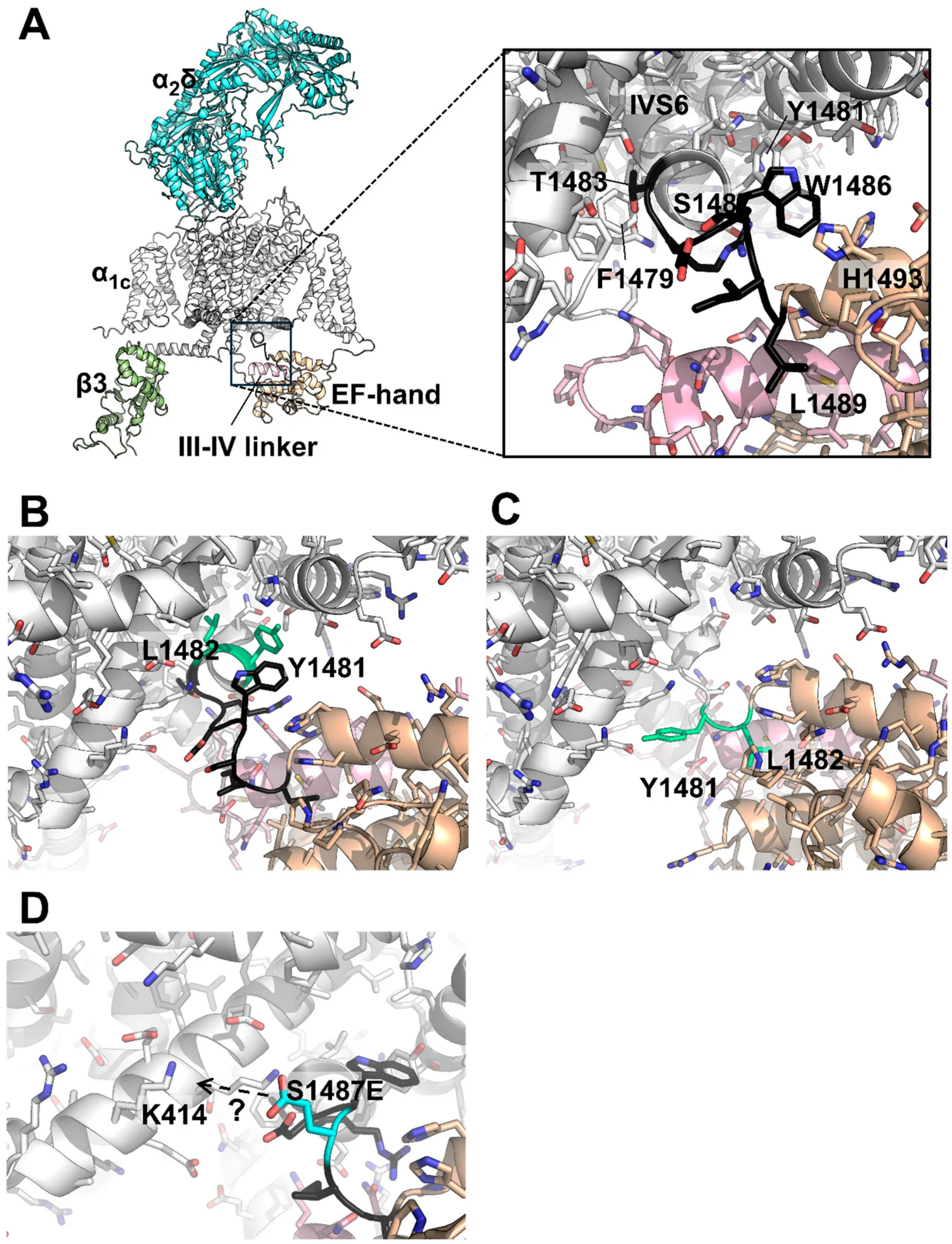
**Structural analysis of the effects of the 7aa deletion and S1487E mutations.** (**A**) Cryo-EM structure of human Ca_v_1.2 (pdb: 8EOG) showing the various subunits. The positions of the EF-hand (beige) interacting with the III-IV linker (pink) are shown. The inset shows a close-up of the region, with the 7 amino acids (residues 1483–1489) affected by the deletion in black. Select residues within this linker and surrounding areas are labelled for reference (**B**) Alphafold model of mouse Ca_v_1.2. The interactions are similar to those observed in the experimental structure in panel A. Two residues in the IVS6 segment (Y1481 and L1482) are highlighted in green (**C**) Alphafold model of mouse Ca_v_1.2 lacking the 7 residues affected by the 7aa deletion. This shows that several helical residues in IVS6 need to unwind to provide a new linker, as otherwise the EF-hand domain would clash with the transmembrane region. (**D**) Alphafold model of mouse Ca_v_1.2 containing S1487E mutation (blue).

The 7aa deletion affects seven residues (TRDWSIL) that encode a linker (1483–1489) connecting the IVS6 helix and the EF-hand domain (Figure 2A). Structural mapping using the available cryo-EM structure of human Ca_v_1.2 shows that this region extensively interacts with residues in both IVS6 and the EF-hand domain [21]. Notably, T1483 is located near the IVS6 and IS6 interface. W1486 interacts with Y1481 in IVS6, whereas L1489 is part of a hydrophobic core with the EF-hand domain. Thus, removing these residues is predicted to significantly destabilize both the EF-hand domain and S6 helices interactions. However, deletion of the entire linker also disrupts the location of the EF-hand in relation to IVS6. The only way to avoid the resulting structural clashes is by unwinding the helical regions to create a new linker. To test this, we compared Alphafold3 [22] models of mouse Ca_v_1.2 with and without the linker. In this model, the terminal residues Y1481 and F1482 unwind, abolishing a range of interactions. An alternative would be unwinding of the first helix in the EF-hand domain, but, in either scenario, there would be a major energetic penalty (Figure 2A). Overall, the combined loss of interactions of the original linker residues, along with significant conformational changes, would result in major destabilization whereby the channel misfolds and is likely targeted for degradation.

Modeling of the S1487E mutation suggests that the Glu may form interactions with a Lys on the Domain I S6 helix (Figure 2D). Based on the available structure of Ca_v_1.2, we propose that the S1487E mutation would have minimal functional impact, as there is sufficient space for glutamic acid at this position. The nearest positively charged residue, Lys414, is ~8 Å away, whereas good ionic interactions typically require a distance below 5 Å. Whilst conformational changes could potentially reduce this 8 Å distance, this would involve significant movement of the IS6 helix and/or movement of the EF-hand domain towards one another. Thus, we deduce that the S1487E mutation is unlikely to meaningfully affect Ca_v_1.2 folding. Taken together, based on available structural data of Ca_v_1.2, it is predicted that deletion of seven amino acids in the proximal C-terminal region of the channel may cause channel destabilization, misfolding, and degradation, whereas substitution of serine by glutamic acid at position 1487 has no major impact on Ca_v_1.2 structure.

### 3.3. Mutations in the 7aa Region That Include S1487 in Ca_v_1.2 Have Functional Consequences In Vivo

To fully understand the effect of mutations disrupting the 7aa region surrounding S1487, functional studies were carried out on mice carrying the S1487A^+/+^, S1487E^+/−^, or 7aa del^+/−^ mutations. ECG and echocardiography recordings were obtained from mice up to 52 weeks old (Table 2). The 7aa del^+/−^ and S1487E^+/−^ mice exhibited no changes in ECG parameters compared to wt. S1487A^+/+^ mice, which displayed a slightly higher heart rate and corresponding shortened RR and PR intervals and an unchanged QTc interval (*p* = 0.0002, *p* < 0.0001, *p* = 0.0382, and *p* > 0.9999, respectively) (Table 2).

**Figure 3:**
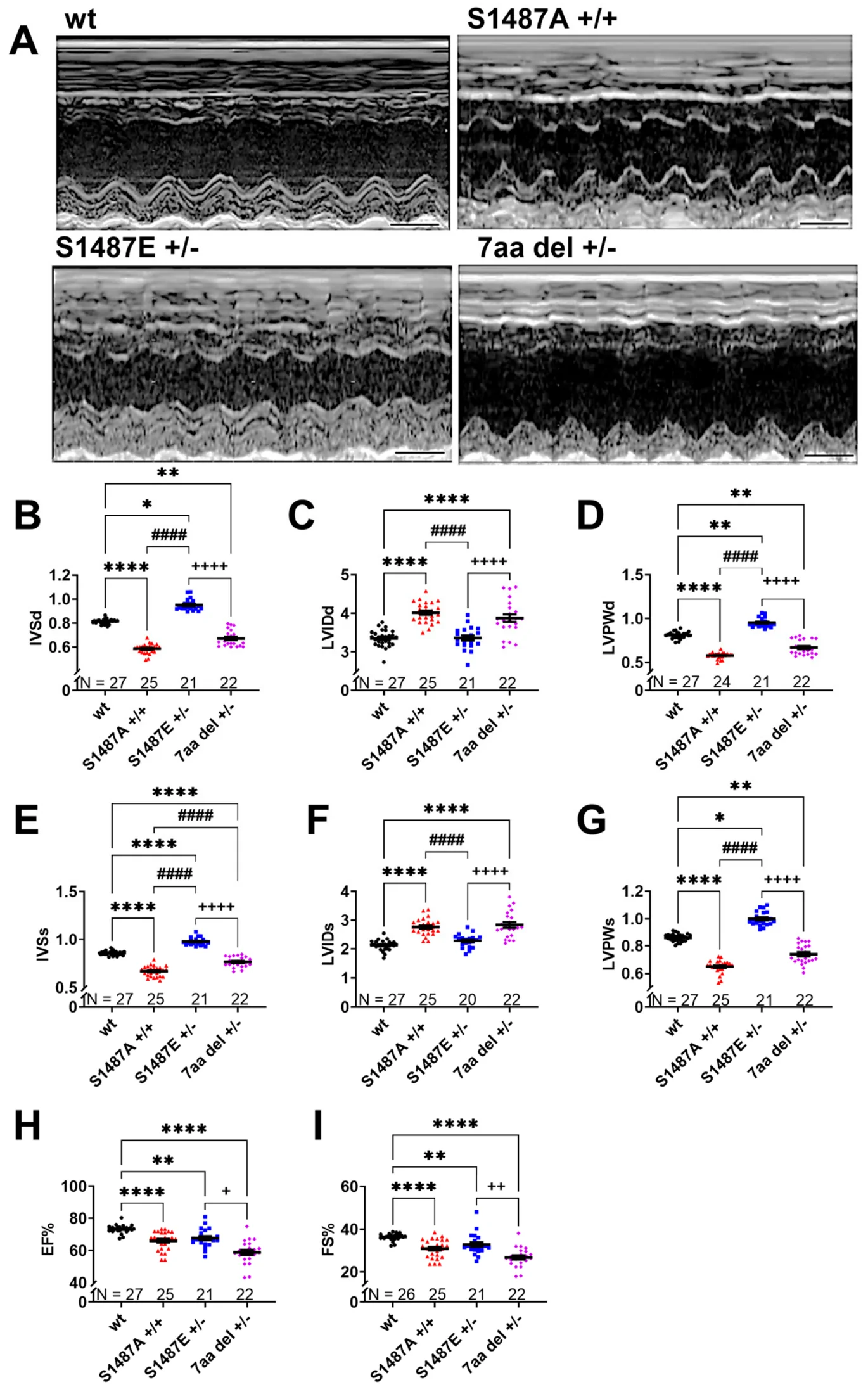
**Mutations in S1487 and deletion of the 7aa region alter echocardiography parameters.** (**A**) Representative echocardiograms. Scale bar = 25 mm/0.125 s. (**B**–**I**) Echocardiography parameters confirm the hypertrophic phenotype associated with S1487E^+/−^ model (panel (**A**) **bottom left**) and slightly dilated 7aa del^+/−^ hearts (panel (**A**) **bottom right**). Data reported as mean ± SEM. Multiple comparisons were performed using one-way ANOVA with Kruskal–Wallis test and Dunn’s post hoc test. Number of mice for each group is labelled within the graphs. Abbreviations: IVSd: interventricular septum thickness during diastole; IVSs: interventricular septum thickness during systole; LVIDd: left ventricular internal diameter during diastole; LVIDs: left ventricular internal diameter during systole; LVPWd: left ventricular posterior wall thickness during diastole; LVPWs: left ventricular posterior wall thickness during systole; EF%: ejection fraction; FS%: fractional shortening (* = vs wt, # = vs S1487A^+/+^, and + = vs 7aa del^+/−^); * *p* < 0.05, ** *p* < 0.01, **** *p* < 0.0001. Symbols denote group comparisons and number of symbols denotes level of significance.

Echocardiography studies revealed a decrease in the interventricular septum thickness (IVS) (Figure 3B,E) and left ventricular posterior wall thickness (LVPW) (Figure 3D,G) in both 7aa del^+/−^ and S1487A^+/+^ models compared to wt. Thinning of the left ventricular wall and increased internal diameter (LVID) (LVIDd: *p* < 0.0001; LVIDs: *p* < 0.0001 in both) (Figure 3C,F) indicate a dilated phenotype (IVSd: *p* = 0.0061; IVSs: *p* < 0.0001; LVPWd: *p* = 0.0074; LVPWs: *p* = 0.0071). Both S1487A^+/+^ and 7aa del^+/−^ mutations resulted in reduced cardiac contractility observed as decreased fractional shortening (FS%) and ejection fraction (EF%) (FS%: *p* < 0.0001; EF%: *p* < 0.0001) (Figure 3H,I). As a result, the higher heart rate at baseline could be a compensatory mechanism for the mild dilated cardiac phenotype we observed in S1487A^+/+^ and 7aa del^+/−^ mutant mice.

Consistent with the increase in THW/TL measurements in S1487E^+/+^ neonatal pups and S1487E^+/−^ adult mice, an increase in IVS (Figure 3B,E) and LVPW was recorded on echocardiography (Figure 3D,G) (IVSd: *p* = 0.0138; IVSs: *p* < 0.0001; LVPWd: *p* = 0.0088; LVPWs: *p* = 0.011) compared to wt, confirming the presence of cardiac hypertrophy. In addition, there was a decrease in the EF% (Figure 3H) and FS% (Figure 3I), indicating abnormal and reduced contractility in these hearts (EF%: *p* = 0.0032, FS%: *p* = 0.0096). No changes were observed in the LVID in S1487E^+/−^ mice (Figure 3C,F).

Overall, assessment of baseline parameters confirmed that Ca_v_1.2 mutations targeting the 7aa region result in alterations in contractility measured on echocardiography and electrophysiology measured on ECG.

### 3.4. NFAT3 and GATA4 Signaling Pathways Induce Cardiac Hypertrophy in S1487E^+/−^ Mutant Mice

**Figure 4:**
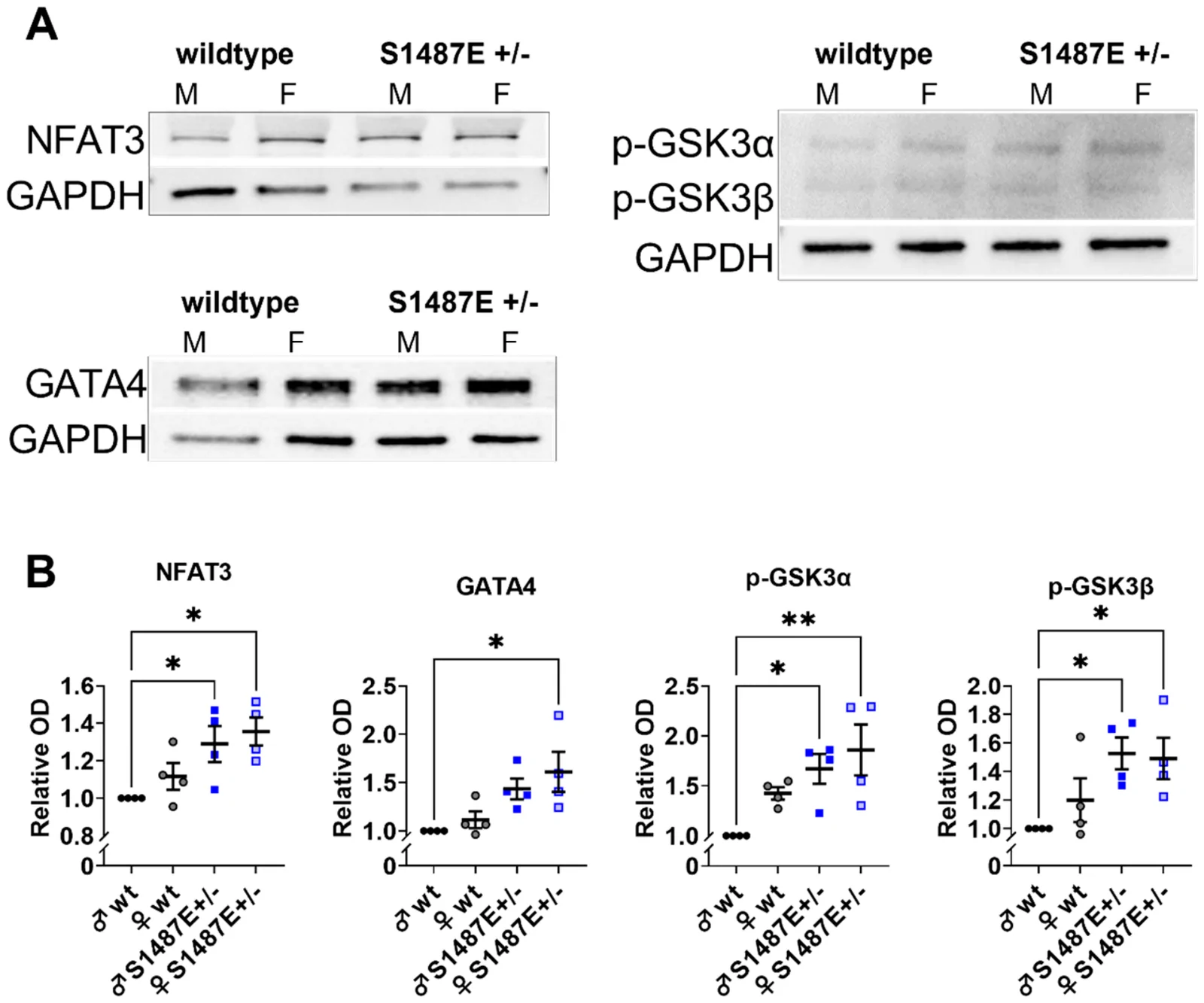
**NFAT3, GATA4, and GSK3 pathways are responsible for hypertrophy observed in S1487E^+/^^−^ mutant model.** (**A**) Representative Western blots and (**B**) quantitative analysis as mean ± SEM optical density on densitometer shown for each signaling pathway. Statistical analysis using one-way ANOVA with Dunnett’s post hoc test. * *p* < 0.05, ** *p* < 0.01.

Following confirmation of cardiac hypertrophy in S1487E^+/−^ mutant mice on echocardiography, we investigated key regulators of cardiac signaling (Figure 4). NFAT3 is a downstream regulator of calcineurin signaling, which is responsible for cardiac development and growth [23]. NFAT3 interacts with other regulatory components such as GATA, which can induce hypertrophy [23]. Both NFAT3 and GATA4 were elevated in hearts of S1487E^+/−^ mice when compared to controls (Figure 4B). This confirms the involvement of the NFAT-GATA pathway in S1487E-mediated hypertrophy. Subsequently, we investigated involvement of downstream regulators of NFAT and GATA4. We found that glycogen synthase kinase 3 (GSK3), a known downstream regulator of NFAT and GATA4, is upregulated as the active phosphorylated GSK (p-GSK3) [24]. Our data indicate that constitutive activation of S1487 by glutamic acid substitution promotes NFAT-GATA4 hypertrophic signaling pathways, resulting in cardiac hypertrophy in these mice.

### 3.5. Only S1487A^+/+^ Mutation in Murine Ca_v_1.2 Blunted the Response to β-Adrenergic Receptor Stimulation In Vivo

Increased calcium influx through Ca_v_1.2 following β-adrenergic receptor stimulation is characteristic of the “Fight or Flight” response. As S1458 was previously identified as critical in regulating this pathway, we aimed to investigate this in vivo [10]. Our results indicate that the residues in the 7aa region, including S1487, are essential for development (Figure 1E), channel structure and folding (Figure 2), and cardiac contractility (Figure 3). Consequently, we assessed the role of this region during β-adrenergic receptor stimulation in vivo. We exposed mice to a single intraperitoneal injection of 20 mg/kg of isoproterenol (ISO) and assessed changes in ECG and echocardiography parameters. We calculated fold changes in response to acute ISO treatment in order to highlight the effect of each mutation on the responsiveness to β-adrenergic receptor stimulation. For each parameter, a fold change of 1 represents no change, fold change > 1 indicates an increase, and fold change < 1 indicates a decrease.

**Figure 5:**
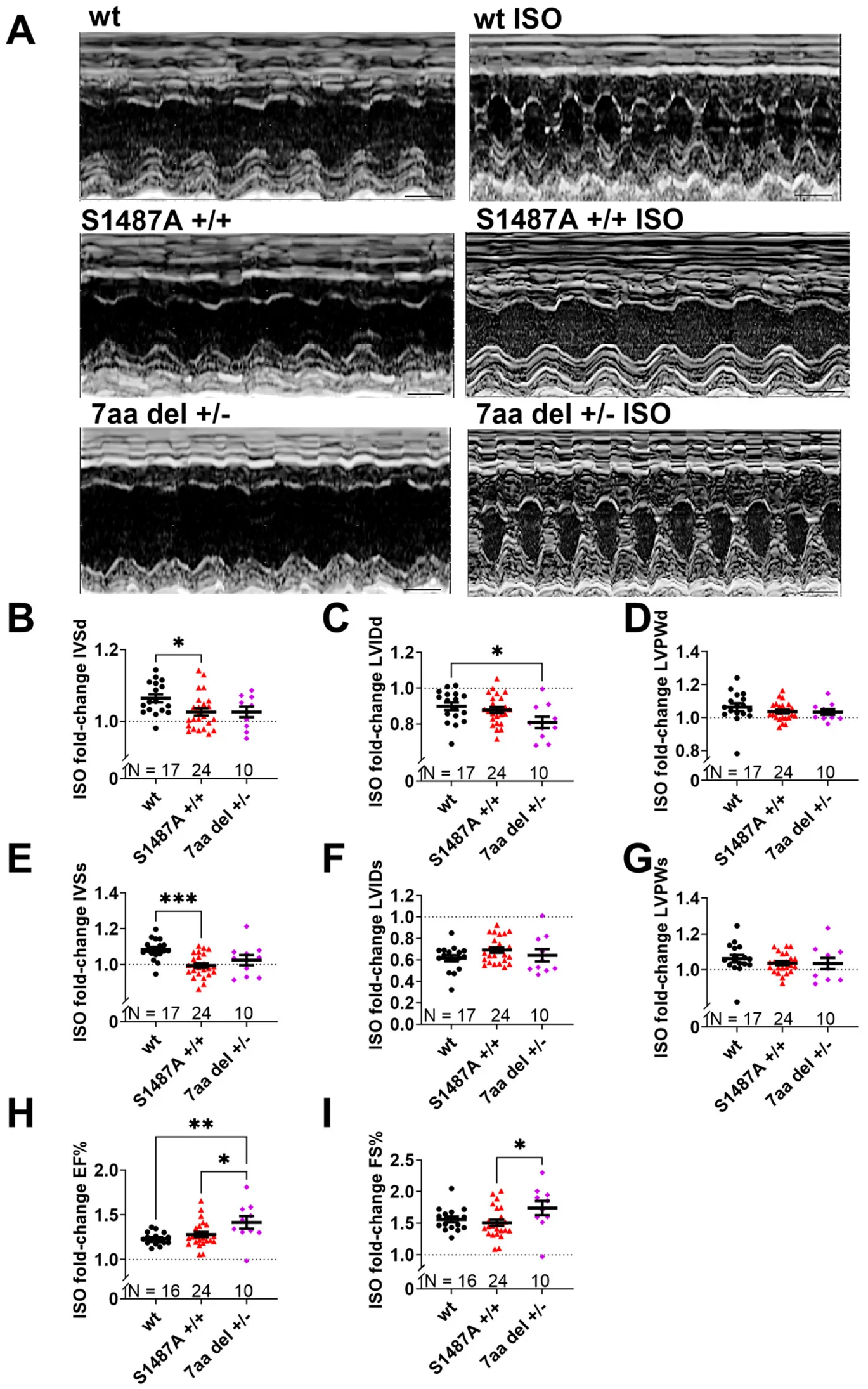
**S1487A^+/+^ mice are less responsive to acute β-adrenergic receptor stimulation.** (**A**) Representative echocardiograms. Scale bar = 25 mm/0.125 s. (**B**–**I**) Echocardiography results depicting the fold change in each parameter in response to acute β-adrenergic receptor stimulation through treatment with ISO. Multiple comparisons were performed using one-way ANOVA with Kruskal–Wallis test and Dunn’s post hoc test. Numbers of mice for each group are labelled within the graphs. Abbreviations: IVSd: interventricular septum thickness during diastole; IVSs: interventricular septum thickness during systole; LVIDd: left ventricular internal diameter during diastole; LVIDs: left ventricular internal diameter during systole; LVPWd: left ventricular posterior wall thickness during diastole; LVPWs: left ventricular posterior wall thickness during systole; EF%: ejection fraction; FS%: fractional shortening. * *p* < 0.05, ** *p* < 0.01, *** *p* < 0.001.

Stimulation of β-adrenergic receptors with ISO led to an increase in the heart rate in wt and 7aa del^+/−^ mice, consistent with a response by the unaffected allele of the channel in 7aa del^+/−^ mice (Table 2). Echocardiography analysis revealed a blunted ISO-induced increase in IVS in S1487A^+/+^ mice compared to wt (IVSd: *p* = 0.0404, IVSs: *p* = 0.0004) (Figure 5B,E). In diastole, a greater decrease in LVID was measured in 7aa del^+/−^ mice compared to wt (*p* = 0.0387) (Figure 5C). There were no changes to LVPW in response to acute β-adrenergic receptor stimulation (Figure 5D,G). Contractility was increased in 7aa del^+/−^ mice following acute β-adrenergic receptor stimulation, assessed as changes in EF% and FS% (Figure 5H,I). The 7aa del^+/−^ mutants exhibited acute β-adrenergic responses comparable to that of wt. Our structural analysis (Figure 2) indicates that deletion of the region surrounding serine 1487 has a high probability of causing misfolding of the mutant protein. As we did not measure any differences in channel protein expression (Supplementary Figure S5), we hypothesize that functional Ca_v_1.2 channel proteins are formed by the expression of the unaffected wt allele, probably at a lower copy number, along with the mutant channel protein with altered function.

Our in vivo data highlight the importance of S1487 in the regulation of the “Fight or Flight” response. When S1487 is rendered inaccessible to cAMP-dependent PKA phosphorylation by substitution with an alanine residue, the β-adrenergic response is blunted, as shown by the overall reduced response to acute ISO treatment in S1487A^+/+^ mutant mice.

### 3.6. S1487A^+/+^ Mutant Mice Are Less Responsive to Chronic β-Adrenergic Receptor Stimulation Compared to wt Mice

**Figure 6:**
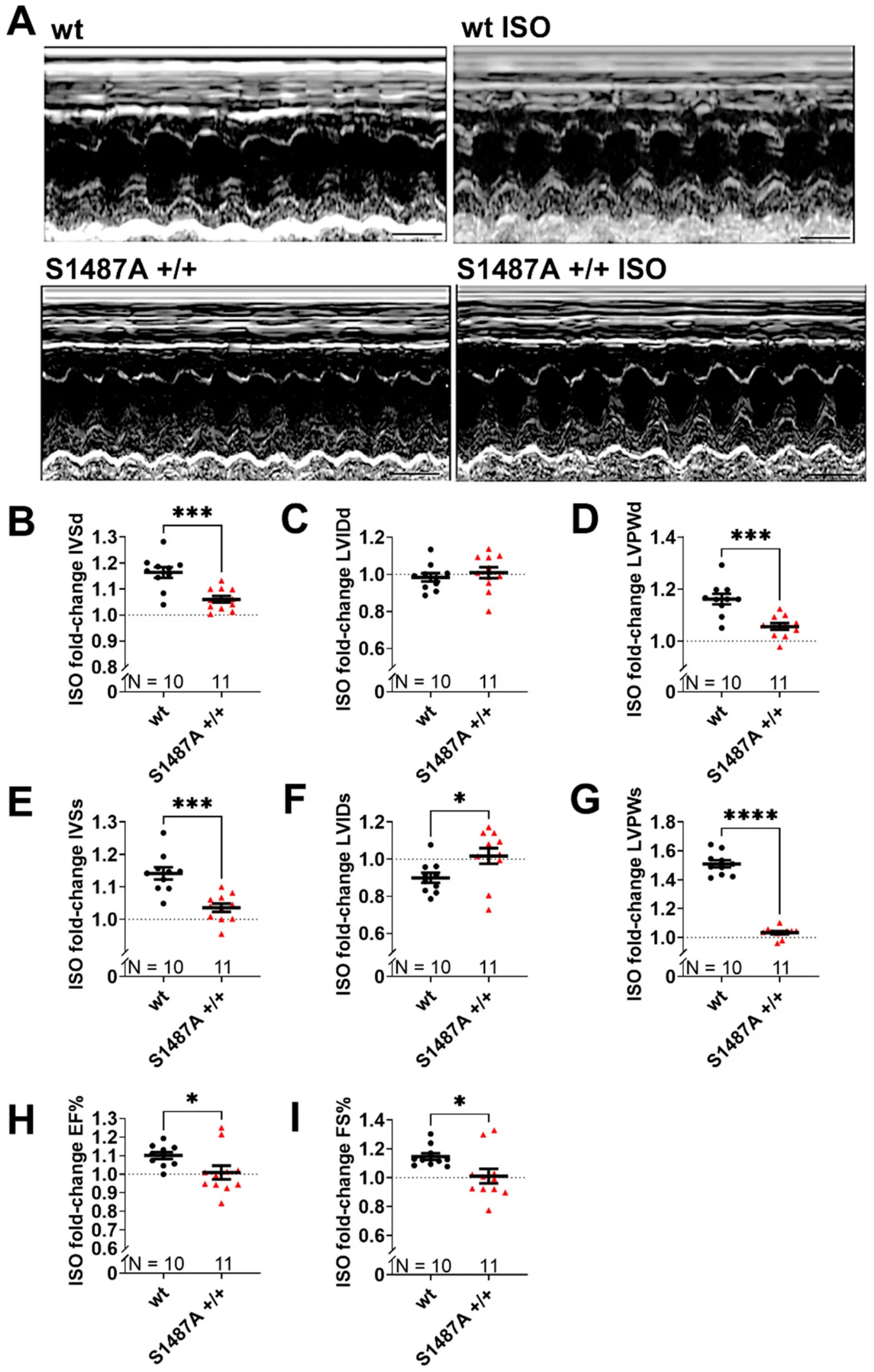
**S1487A^+/+^ mice are less responsive to chronic β-adrenergic receptor stimulation.** (**A**) Representative echocardiograms. Scale bar = 25 mm/0.125 s. (**B**–**I**) Echocardiography results depicting the fold change in each parameter in response to chronic β-adrenergic receptor stimulation following treatment with ISO (**A**–**H**). Multiple comparisons were performed using one-way ANOVA with Kruskal–Wallis test and Dunn’s post hoc test. Number of mice for each group are labelled within the graphs. Abbreviations: IVSd: interventricular septum thickness during diastole; IVSs: interventricular septum thickness during systole; LVIDd: left ventricular internal diameter during diastole; LVIDs: left ventricular internal diameter during systole; LVPWd: left ventricular posterior wall thickness during diastole; LVPWs: left ventricular posterior wall thickness during systole; EF%: ejection fraction; FS%: fractional shortening. * *p* < 0.05, *** *p* < 0.001, **** *p* < 0.0001.

To examine the effects of chronic β-adrenergic receptor stimulation, both wt and S1487A^+/+^ mice were administered intraperitoneal injections of 20 mg/kg of ISO daily for 2 weeks. Analysis of ECG parameters indicated that S1487A^+/+^ mice developed a reduced heart rate in response to chronic ISO (*p* = 0.02) and a corresponding increase in the RR interval (*p* = 0.0257) compared to wt (Table 2). Echocardiography analysis indicated minimal effect of chronic ISO in S1487A^+/+^ mice, as most parameters exhibited a mean fold change of 1. Hearts expressing the wt Ca_v_1.2 gene recorded an increase in IVS (IVSd: *p* = 0.004; IVSs: *p* = 0.001) (Figure 6A,D) and LVPW (LVPWd: *p* = 0.002; LVPWs: *p* < 0.0001) (Figure 6C,F) and a decrease in the LVIDs (*p* = 0.0337) (Figure 6E) compared to the S1487A^+/+^ model, consistent with the development of ISO-induced hypertrophy. These data show that the structural effects of chronic β-adrenergic receptor stimulation in the wt heart are not observed in hearts expressing the S1487A^+/+^ mutation. In addition, the EF% (*p* = 0.0242) and FS% (*p* = 0.0101) were significantly increased in wt mice after chronic β-adrenergic receptor stimulation compared to the S1487A^+/+^ (Figure 6G,H).

Altogether, we demonstrate a blunted effect in S1487A^+/+^ mutant mice compared to wt mice after chronic β-adrenergic receptor stimulation.

### 3.7. β-Adrenergic Receptor Stimulation Alters I_CaL_ Kinetics and Calcium Transients in wt but not S1487A^+/+^, S1487E^+/−^, or 7aa Del^+/−^ Cardiac Myocytes

**Figure 7:**
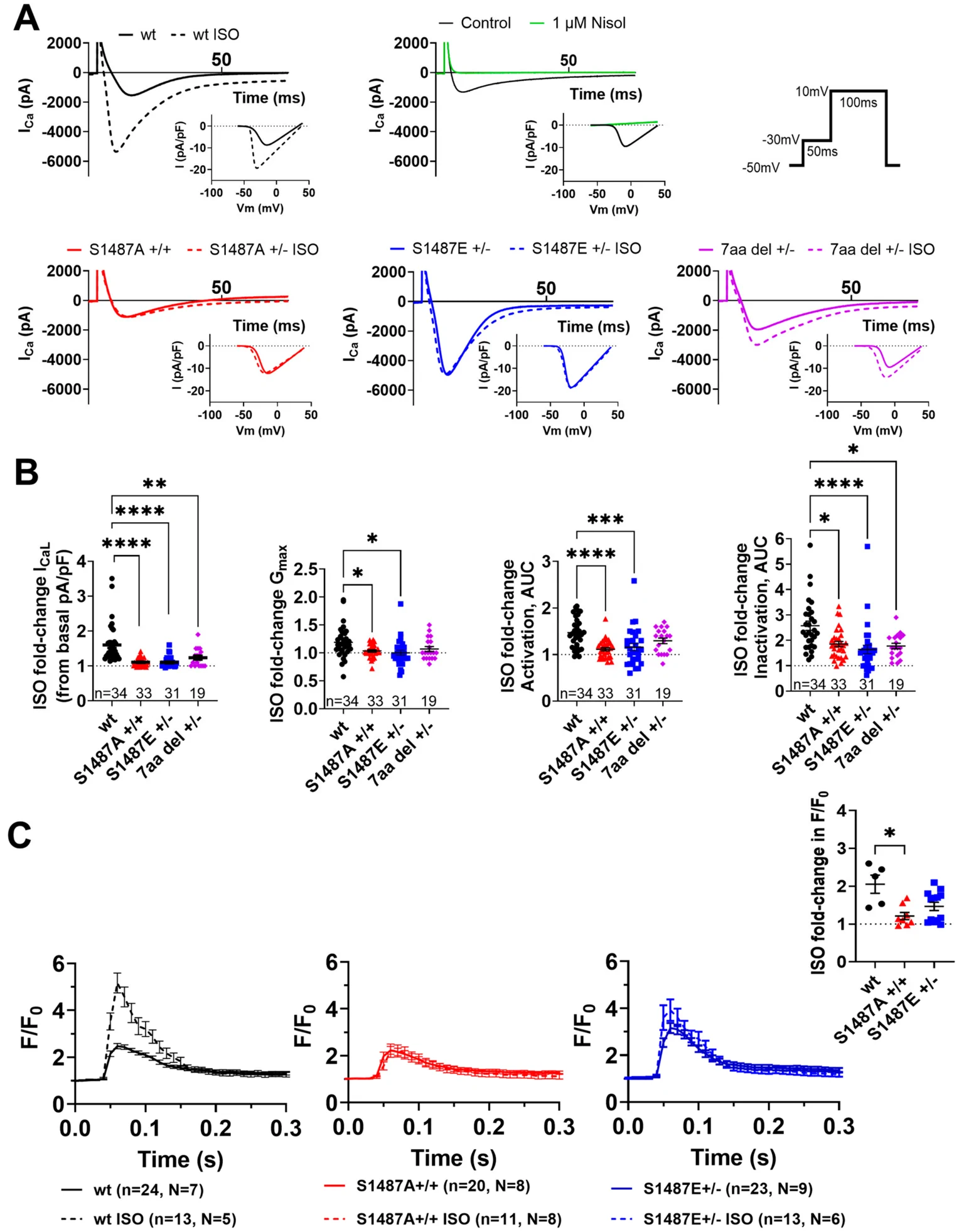
**β-adrenergic receptor activation alters Ca_v_1.2 kinetics and calcium transients in wt but not S1487A^+/+^, and S1487E^+/^^−^ cardiac myocytes.** (**A**) Representative I_CaL_ traces and I/V curves (inset) before and after ISO and proof of Ca_v_1.2 current by Nisoldipine. (**B**) Fold change from basal in I_CaL_ and biophysical parameters that occurred following exposure to ISO. Mean ± SEM; comparisons were performed using one-way ANOVA with Kruskal–Wallis test with Dunn’s post hoc test. *n* = no. of cardiomyocytes (**C**) Analysis of Fluo-4 fluorescence in myocytes from wt, S1487A^+/+^, and S1487E^+/−^ hearts’ average calcium transients elicited with Fluo-4 in wt, S1487A^+/+^, and S1487E^+/−^ mutant cardiomyocytes (*N* = no. of mice, *n* = no. of cardiomyocytes) and relative increase in F/F_O_ (presented as mean ± SEM) (inset); * *p* < 0.05, ** *p* < 0.01, *** *p* < 0.001, **** *p* < 0.0001.

Our in vivo analysis of mutations targeting this 7aa region demonstrates the importance of this site in regulating channel function and the β-adrenergic response. To gain more insight into these effects, cardiac myocytes were isolated from adult mice expressing S1487A^+/+^, S1487E^+/−^, and 7aa del^+/−^ mutations or wt channel. Electrophysiological parameters were assessed at baseline and after acute exposure to 100 nM ISO. The S1487E^+/−^ mutant cardiomyocytes exhibited an increased basal I_CaL_ compared to wt (wt: −8.36 ± 0.67 pA/pF; S1487E^+/−^: −12.11 ± 1.00 pA/pF, *p* = 0.0121), consistent with constitutive activation of the channel. The wt and 7aa del^+/−^ cardiomyocytes recorded an increased I_CaL_ after acute β-adrenergic receptor stimulation, as measured by the fold increase, although this was higher in wt cells (wt: 1.6 ± 0.09; 7aa del^+/−^: 1.3 ± 0.06; *p* = 0.0383). In contrast, there was minimal ISO-induced increase in I_CaL_ observed in either S1487A^+/+^ or S1487E^+/−^ mutant myocytes compared to wt (Figure 7A). Analysis of additional parameters demonstrated an ISO-induced increase in several parameters in wt cardiomyocytes, including channel conductance (G_max_), the area under the curve (AUC) of activation, and the AUC of inactivation (Figure 7C). These responses were significantly greater in wt cells compared to cells expressing the S1487A^+/+^ and S1487E^+/−^ mutations. Although there was a small increase in the AUC of inactivation of wt cells compared to cells expressing the 7aa del^+/−^ mutation, the G_max_ and AUC of activation remained comparable to wt cells. We performed calcium transients using Fluo-4 dye (Figure 7C). There was a significantly greater increase in the amplitude of calcium transients in wt cells compared with that of the S1487A^+/+^ cells (*p* = 0.0226). ISO did not further increase the calcium transient in S1487A^+/+^ cells or in S1487E^+/−^ myocytes.

Some of the 7aa del^+/−^ mutant cells responded to ISO, most likely attributed to the presence of wt channel protein. However, the lack of a response in cardiomyocytes expressing S1487A^+/+^ following β-adrenergic receptor stimulation in vitro was striking.

### 3.8. Absence of β-Adrenergic Responses in a Heterologous Expression System of Ca_v_1.2

The functional importance of phosphorylation at S1487 was next tested in a heterologous expression system. We expressed α1C, α2δ, and β2b subunits of Ca_v_1.2 in HEK293T cells. Previous studies have demonstrated that the presence of a small G protein, Rad, bound to Ca_v_1.2 inhibits calcium influx and is necessary for regulation of the β-adrenergic response [25,26,27]. To test its involvement in this response in our S1487A model, we co-expressed Rad in HEK cells expressing wt- or S1487A-Ca_v_1.2. The effect of PKA phosphorylation was then assessed by recording barium currents (I_Ba_) following stimulation with forskolin (Frsk).

**Figure 8:**
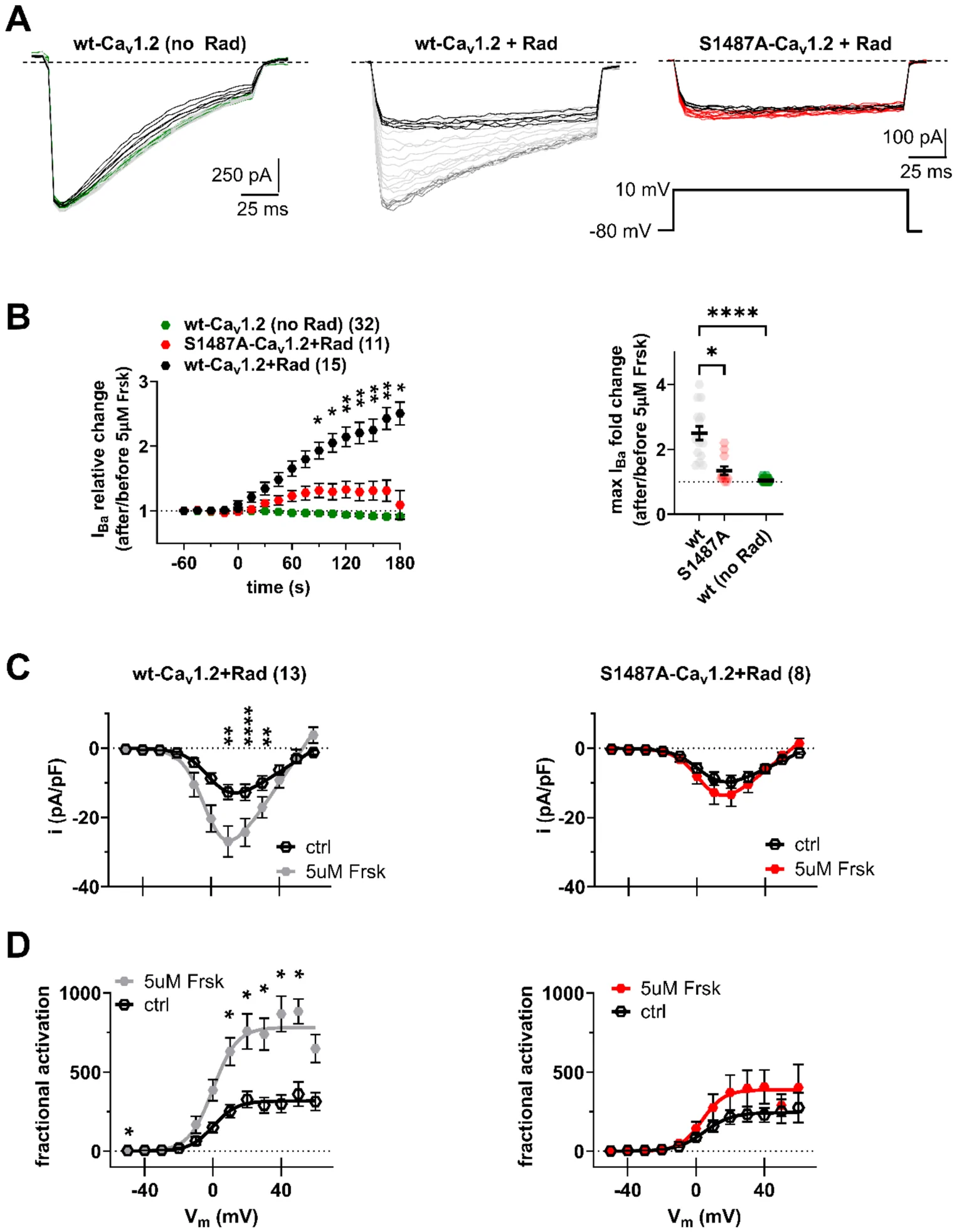
**PKA phosphorylation at S1487 is necessary for increased I_Ba_ through the Ca_v_1.2 channel.** (**A**) Representative I_Ba_ elicited through step depolarization to 10 mV from the holding potential of −80 mV recorded before/after forskolin addition in wt-Ca_v_1.2 (no Rad) (**left**), wt-Ca_v_1.2+Rad (black/gray) (**middle**), and S1487A-Ca_v_1.2+Rad (dark red/red) (**right**) in heterologous expressed channels in HEK cells. (**B**) (**left**) Time courses of I_Ba_ fold changes before and after 5 μM forskolin addition. Forskolin was added at time 0 s. Two-way ANOVA mixed effect followed by Šídák’s multiple comparisons test. (**right**) Corresponding forskolin-induced I_Ba_ fold changes. Unpaired two-way Mann–Whitney *t*-test. (**C**) I/V curves and corresponding fractional activations. (**D**) All parameters represented in wt-Ca_v_1.2+Rad (**left**) and S1487A-Ca_v_1.2+Rad (**right**) and all recorded before and after 5 μM forskolin addition. Data are represented as mean ± SEM. Two-way ANOVA followed by Sidák’s multiple comparisons test and paired 2-tailed *t*-test; * *p* < 0.05, ** *p* < 0.01, **** *p* < 0.0001. No. of cells studied is shown in brackets.

We demonstrate that I_Ba_ significantly increased following exposure to Frsk in wt-Ca_v_1.2+Rad expressing cells. However, the Frsk response in cells expressing S1487A-Ca_v_1.2+Rad was significantly attenuated compared to wt-Ca_v_1.2+Rad cells (Figure 8A right). Quantification of the fold increase in I_Ba_ following exposure to Frsk revealed that the relative fold increase of I_Ba_ for cells expressing S1487A-Ca_v_1.2+Rad is significantly less than wt-Ca_v_1.2+Rad (Figure 8B). The increase in I_Ba_ was also quantified at the maximal peak current, which confirmed a reduced response in S1487A-Ca_v_1.2+Rad transfected cells (1.35 ± 0.13) compared to wt-Ca_v_1.2+Rad (2.5 ± 0.21 *p* = 0.0001) (Figure 8B). The current/voltage (I/V) relationship demonstrated a significant increase in I_Ba_ between 0 and 20 mV during exposure to Frsk in wt-Ca_v_1.2+Rad cells (Figure 8C). In contrast, no changes were observed for S1487A-Ca_v_1.2+Rad (Figure 8C right). Analysis of fractional activation shows a significant increase with Frsk in wt-Ca_v_1.2+Rad cells but no change in S1487A-Ca_v_1.2+Rad (Figure 8D). Maximal conductance (*G_max_*) of wt-Ca_v_1.2+Rad increased by 2.2-fold, and there was a hyperpolarizing shift of wt-Ca_v_1.2+Rad activation midpoint (*V*_1/2_) and a decrease of the slope of the channel activation (k_act_) following exposure to Frsk (Table 3). There were no changes in the activation parameters of S1487A-Ca_v_1.2+Rad. The paired comparison of I_Ba_ parameters before and after Frsk revealed that in wt-Ca_v_1.2+Rad, peak I_Ba_ was increased, I_Ba_ density was augmented, and there was greater AUC of activation, whereas none of these changes were observed in S1487A-Ca_v_1.2+Rad (Table 3). Taken together, S1487 plays a significant role in the functional regulation of Ca_v_1.2. The S1487A mutation significantly prevented I_Ba_ augmentation following Frsk application. Therefore, S1487A-Ca_v_1.2+Rad resulted in diminished I_Ba_, and no alterations in *I*/*V* curves or I_Ba_ properties before or after Frsk application.

## 4. Discussion

The physiological reaction to a perceived threat is an important evolutionary survival mechanism, and Ca_v_1.2 plays a critical role in increasing heart rate and cardiac output in the “Fight or Flight” response. Despite this, the molecular mechanisms responsible continue to be debated. In a previous study, we identified a novel PKA phosphorylation site, S1458, that was essential for the β-adrenergic response in vitro [10]. Mutating S1458A rendered the site inaccessible to phosphorylation. This was functionally relevant, as exposure of the purified short NT truncated C-terminus form of Ca_v_1.2 containing S1458A to PKA did not induce a further increase in open channel probability. These data informed our current study, where we aimed to validate the importance of S1458 and the 21 bp region in a series of in vivo mouse models, targeting the mouse homologue at S1487.

### 4.1. S1487A^+/+^ Mice Demonstrate Blunted Responses to β-Adrenergic Receptor Stimulation

Consistent with our in silico modeling, S1487A^+/+^ mice were viable with a mild phenotype and provided a tool to assess the effects of a phosphonull mutation of the PKA site in vivo. Mature mice displayed altered electrophysiology and echocardiography parameters at baseline, consistent with mild ventricular dilatation and reduced contractility. We measured Ca_v_1.2 and Rad expression in S1487A^+/+^ hearts and found no differences in expression compared to wt (Supplementary Figure S3). However, when the mice were challenged with ISO in vivo, no further increases in contractility were recorded, nor did they develop hypertrophy, as we observed in wt mice. Some increase in the heart rate of S1487A^+/+^ mice occurred, as other ion channels (I_f_ and I_CaT_) involved in pacemaking at the sinoatrial node are unaffected by the mutation and therefore remain responsive to adrenergic activation. Our in vitro results further confirmed a lack of response to ISO in S1487A^+/+^ ventricular cardiomyocytes. In addition, activation and inactivation kinetics and channel conductance were also unaffected by ISO in the mutant cells when compared to wt. These data validate S1487 as a functionally important PKA phosphorylation site.

### 4.2. S1487E^+/−^ Mice Develop Hypertrophy

If S1487 plays a pivotal role in β-adrenergic receptor stimulation of Ca_v_1.2, then mutation to glutamic acid should mimic a physiological responses consistent with constitutive activation of the site. Strikingly, we found that S1487E^+/+^ mice were embryonic lethal, providing further evidence for the importance of S1487 in Ca_v_1.2 function and also in embryonic development. Post mortem analysis of perinatal hearts revealed significant hypertrophy, confirming severely impaired Ca_v_1.2 function in S1487E^+/+^ mice, which was incompatible with postnatal life. Our findings were supported by the predicted score of “likely pathogenic” assigned to this mutation by AlphaMissense software [19]. Adult S1487E^+/−^ mice developed significant hypertrophy compared to wt, further highlighting the severity of this phenotype. This was evident in baseline echocardiography recordings, as an increase in the thickness of the IVS and LVPW and a decrease in the LVID. While the precise proportion of functional channels containing the mutation is unclear, it is not necessary for all channels to be constitutively active to produce a measurable phenotype. This is supported in the previous literature, where heterozygote Cacna1c knockout mice were found to exhibit cardiac hypertrophy, ventricular dilation, and heart failure [28]. In addition, Blaich et al. reported that homozygote mutation of residue 1624 in the IQ motif led to embryonic lethality, with heterozygosity resulting in dilated cardiomyopathy, fibrosis, and cell death [29]. We confirmed that Ca_v_1.2 and Rad expression were unaffected by the S1487E mutation and thus not responsible for the hypertrophic phenotype observed (Supplementary Figure S3). However, we measured an increase in GATA4 and NFAT3 signaling and increased phosphorylation of GSKα and GSKβ. Constitutive activation of this site was confirmed in vitro, as we recorded significantly increased basal I_CaL_ compared to wt in cardiac myocytes from hearts of S1487E^+/−^ mice in addition to a lack of response to ISO (Figure 7A,B,D).

### 4.3. A 7aa Region in the Proximal C-Terminus of Ca_v_1.2 Is Critical for Viability and Proper Development

In this study, we confirm that a 7aa region in the proximal C-terminus of the cardiac Ca_v_1.2 channel is an important regulator of Ca_v_1.2 function and β-adrenergic signaling. AlphaMissense predictions in this region were predominantly “likely pathogenic”. Consistent with this, 7aa del^+/+^ mice were embryonic lethal. Adult 7aa del^+/−^ mice developed a dilated phenotype, which manifested as decreased IVS and LVPW, increased LVID, and decreased EF% consistent with impaired contractility. Acute exposure of 7aa del^+/−^ mice to ISO led to minimal differences in contractility compared to wt, which included a small decrease in LVID and increased EF% and FS%. Our in vitro studies in cardiomyocytes can be similarly explained by the nature of this genotype, as there was a reduced effect of ISO in 7aa del^+/−^ myocytes on I_CaL_ and AUC of inactivation when compared to wt. This diminished response to ISO in our 7aa^+/−^ model in vivo and in vitro may be associated with the effects of ISO on the wt channel expressed in the heterozygote phenotype or the absence of Ser1487 in one of the CACNA1C alleles.

### 4.4. Co-Expression of Rad Does Not Alleviate the Effect of S1487A^+/+^ Mutation on the β-Adrenergic Response in a Heterologous Expression System

A small G protein Rad has been implicated in the β-adrenergic regulation of Ca_v_1.2 [9,30,31]. Under baseline conditions, Rad is bound to the Ca_v_1.2 β-subunit, blocking calcium influx. Phosphorylation of Rad after stimulation of β-adrenergic receptors allows it to detach from Ca_v_1.2, facilitating calcium influx characteristic of the β-adrenergic response. We tested whether the introduction of the S1487A or S1487E mutations led to changes in Ca_v_1.2 or Rad expression. Both proteins were conserved in the mutant models, confirming that the effects of the S1487A and S1487E mutations on β-adrenergic responses were occurring independently of changes in Ca_v_1.2 or Rad expression (Supplementary Figure S3). To further assess a role for Rad, we co-expressed α2δ, β2b, and α1C subunits of Ca_v_1.2 and Rad in HEK293T cells. We assessed β-adrenergic responses in cells expressing wt and S1487A^+/+^ forms of the Ca_v_1.2 channel with Rad. After exposure to Forskolin, the β-adrenergic response was significantly attenuated in S1487A^+/+^ cells with Rad. These data clearly demonstrate that S1487 plays a significant role in the β-adrenergic response. Rad phosphorylation and the corresponding β-adrenergic response are linked to binding of the β-subunit to the α1C I–II loop, which facilitates insertion of the channel in the plasma membrane [25]. Mapping S1487 on available cryo-EM structures of Ca_v_1.2 shows that it is close to the I-II loop, and we hypothesize that its phosphorylation or mutation to glutamic acid may cause conformational changes that allow it to form ionic interactions with a Lys in the I-II loop. This may help explain our observation that the β-adrenergic response in HEK293T cells expressing S1487A^+/+^ is strongly attenuated, even in the presence of Rad.

The rabbit homologue of S1487 (S1517) has been investigated previously and was not found to be a crucial regulator of β-adrenergic signaling [9]. However, it must be noted that the experimental approach differed from our studies. The authors replaced 51 serine/threonine residues in the rabbit α1C with alanine at 35 potential PKA phospho-regulatory domains in the 35-mutant α1C construct. They also ligated 3× Flag epitopes in-frame to the *N* terminus and substituted T1066Y and Q1070M to create a dihydropyridine (DHP)-insensitive α1C subunit. They used double-transgenic mice by breeding transgenic mice with non-targeted insertion of these tetracycline-regulated cDNAs with cardiac-specific (α-MHC), doxycycline-regulated, codon-optimized reverse transcriptional transactivator mice. This resulted in transgene expression that did not require doxycycline due to low basal binding of rtTA protein to the Tet operator sequences, indicating that the mutation was always on or activated [26]. While transgenic models are powerful tools for in vivo assessment of mutations, they have limitations. Such a massive mutation strategy could lead to structural and functional alterations (local changes in charge, altered protein folding, dihydropyridine sensitivity, gating dynamics, alterations in regulatory mechanisms), which could prevent examination of whether and how these α1C mutants contribute to both basal and sympathetic regulation of cardiac contractility in vivo [32]. Since the discovery of Rad, others have measured direct adrenergic modulation of voltage-gated Ca^2+^ currents [33], suggesting Rad is not absolutely required for the response but facilitates channel opening following PKA phosphorylation. In addition, Rad gene knockout mice are viable [31], whereas in the present study S1487E^+/+^ and 7aa del^+/+^ mutations were embryonic lethal, further highlighting the importance of S1487 in regulating cardiac function.

In the present study, we used CRISPR-Cas9 technology to investigate the effect of S1487A^+/+^, S1487E^+/−^, and 7aa del^+/−^ mutations on β-adrenergic signaling in vivo, including developmental implications from birth. Extensive phenotyping revealed that S1487E^+/+^ and 7aa del^+/+^ genotypes were not compatible with life, emphasizing the importance of correct folding of the proximal C terminal region of the channel on function. Our study has validated that a serine at 1487 in mouse (1458 in human short NT isoform) Ca_v_1.2 is required for β-adrenergic responses in a 7aa region that is critical to Ca_v_1.2 protein structural integrity and function (Table 4).

## Figures and Tables

**Table 1:** Full list of antibodies used in Western Blot experiments.

Name	Species	Clonality	Isotype	Source	Cat #	Dilution
VDAC	Rabbit	monoclonal	IgG	Cell Signalling	4661S	1:1000–2000
GAPDH	Mouse	monoclonal	IgG2	Cell Signalling	97166S	1:1000–2000
NFAT3 (23E6) (NFATc4)	rabbit	monoclonal	IgG	Cell Signalling	2183S	1:1000
GATA4	rabbit	monoclonal	IgG	Cell Signalling	36966S	1:1000
Phospho-GSK-3β (Ser9) (5B3)	rabbit	monoclonal	IgG	Cell Signalling	9323S	1:1000
Phospho-GSK-3α/β (Ser21/9)	rabbit	polyclonal	IgG	Cell Signalling	9331S	1:1000
CaV1.2	rabbit	polyclonal	IgG	Alomone	ACC-003	1:200
RRAD	goat	polyclonal	IgG	Thermo Fisher	PA5-37885	1:1000

**Table 2:** ECG analysis from baseline and following acute or chronic β-adrenergic receptor stimulation.

*Baseline (Prior to β-AR Stimulation)*	**wt (*N* = 24)**	**S1487A ^+/+^ (*N* = 26)**		**S1487E ^+/−^ (*N* = 20)**		**7aa del ^+/−^ (*N* = 25)**	
	***Mean ± SEM***	***Mean ± SEM***	***p***	***Mean ± SEM***	***p***	***Mean ± SEM***	***p***
**Heart rate (bpm)**	511.33 ± 8.5	569.91 ± 7.9	**0.0002** ^a^**, <0.0001** ^b,c^	509.52 ± 7.1	>0.9999 ^a,c^	509.74 ± 7.9	>0.9999 ^a^
**RR interval (ms)**	118.14 ± 2.1	105.9 ± 1.5	**<0.0001** ^a,b,c^	118.54 ± 1.7	0.9989 ^a^, 0.9837 ^c^	117.56 ± 2.1	0.9959 ^a^
**PR interval (ms)**	51.46 ± 1.4	46.85 ± 1.0	**0.0382** ^a^, **0.0035**^c^	51.19 ± 0.8	0.9989 ^a^, 0.8109 ^c^	52.78 ± 1.5	0.8632 ^a^
**QTc interval (ms)**	41.2 ± 3.12	42.20 ± 2.05	>0.9999 ^a,c,d^	43.13 ± 2.78	>0.9999 ^a,c^	44.56 ± 2.29	>0.9999 ^a^
*Fold change in response to acute β-AR stimulation*	**wt (*N* = 13)**	**S1487A +/+ (*N* = 23)**		**7aa del +/− (*N* = 10)**			
	***Mean ± SEM***	***Mean ± SEM***	***p***	***Mean ± SEM***	***p***		
**Heart rate (bpm)**	1.19 ± 0.02	1.09 ± 0.02	**0.0197** ^a^, 0.1441 ^c^	1.16 ± 0.03	0.8271 ^a^		
**RR interval (ms)**	0.86 ± 0.02	0.91 ± 0.02	0.2065 ^a^, 0.6240 ^c^	0.88 ± 0.03	0.7298 ^a^		
**PR interval (ms)**	0.86 ± 0.02	0.87 ± 0.02	0.9161 ^a^, 0.2884 ^c^	0.93 ± 0.04	0.2676 ^a^		
**QTc interval (ms)**	1.31 ± 0.24	1.08 ± 0.08	>0.9999 ^a,c^	1.01 ± 0.08	>0.9999 ^a^		
*Fold change in response to chronic β-AR stimulation*	**wt (*N* = 8)**	**S1487A +/+ (*N* = 9)**					
	***Mean ± SEM***	***Mean ± SEM***	***p***				
**Heart rate (bpm)**	0.97 ± 0.03	0.87 ± 0.02	**0.0200** ^a^				
**RR interval (ms)**	1.03 ± 0.03	1.15 ± 0.03	**0.0257** ^a^				
**PR interval (ms)**	0.97 ± 0.05	1.10 ± 0.04	0.1139 ^a^				
**QTc interval (ms)**	1.8 ± 0.32	1.21 ± 0.25	0.0511 ^a^				
**a: vs wt, b: vs S1487E +/−, c: vs 7aa del +/−, *N* represents number of animals**							

**Table 3:** Electrophysiological parameters in HEK cells before and after Frsk stimulation. *n* = no. of cells.

	wt-Ca_v_1.2+Rad (*n* = 15)			S1487A-Ca_v_1.2+Rad (*n* = 11)		
	*Baseline*	*Frsk*	*p*	*Baseline*	*Frsk*	*p*
**I_peak_ (pA)**	−248.3 ± 37.4	−542 ± 77.1	**0.0003**	−205 ± 46.25	−286.0 ± 87.93	0.1464
**I (pA/pF)**	−13.6 ± 2.4	−28.54 ± 4.65	**0.0005**	−9.94 ± 2.00	−13.48 ± 3.44	0.1209
**ttp (ms)**	13.95 ± 1.3	12.98 ± 1.06	0.3273	22.35 ± 4.35	26.49 ± 9.85	0.6406
**V_1/2_ (mV)**	1.28 ± 1.4	−0.78 ± 1.94	0.0522	5.18 ± 1.92	6.23 ± 2.3	0.5469
**k_act_ (mV)**	6.33 ± 0.39	4.9 ± 0.34	**0.0003**	7.31 ± 0.66	7.7 ± 0.95	0.7109
**G_max_ (pS/pF)**	18.1 ± 3.5	40.36 ± 6.35	**0.0004**	12.60 ± 3.53	20.85 ± 6.02	**0.0078**
**τ_act_ (ms)**	1.42 ± 0.13	1.83 ± 0.18	**0.0032**	2.16 ± 0.37	2.23 ± 0.28	0.6431
AUC_act_ (pA*ms)	2431 ± 644.0	5218 ± 1240	**0.0005**	4295 ± 1319	4144 ± 1863	0.9453

**Table 4:** Summarized responses of all genotypes.

	=Not Studied		Baseline				Response to β-AR Stimulation			
			In Vivo		In Vitro		In Vivo		In Vitro	
		Viability	ECG	Echo	Myocytes	HEK	ECG	Echo	Myocytes	HEK
**Wildtype**	−/−	**✓ (all)**					response	response	response	response
**S1487A**	**+/**−	**✓**								
	**+/+**	**✓**	↑ HR, ↓ RR, ↓ PR	↓ IVS, ↓ LVPW, ↑ LVID	↑ ttp, ↑ Vrev		response (HR ↑)	no response	no response	no response
**S1487E**	**+/**−	**✓**	=wt	↑ IVS, ↑ LVPW, ↓ LVID	↑ ICa, ↑ Act AUC, ↑ Inact AUC, ↑ Vrev		response (HR ↑)	response	at capacity (phosphormimic)	
	**+/+**	**x**								
**7aa del**	**+/**−	**✓**	=wt	↓ IVS, ↓ LVPW, ↑ LVID	↑ Vrev		no response	reduced response (+/−)	reduced response (+/−)	
	**+/+**	**x**								

## Data Availability

All data will be made available upon request or stored on The University of Western Australia Institutional Research Depository Store IRDS.

## Supplementary Materials

The following supporting information can be downloaded at https://www.mdpi.com/article/10.3390/cells15181655/s1.

## Abbreviations

The following abbreviations are used in this manuscript:

−/−	Wildtype—indicates that neither allele is mutated
+/−	Heterozygote—indicating mutation of one allele
+/+	Homozygote—indicating mutation of both alleles
7aa del	7 amino acid deletion
AKAPs	A kinase anchoring proteins
AUC	Area under the curve
cAMP	Cyclic AMP
DHP	Dihydropyridine
ECG	Electrocardiogram
EF%	Ejection fraction
F	Fluorescence
F_0_	Normalized fluorescence
FBS	Fetal bovine serum
Frsk	Forskolin
FS%	Fractional shortening
G_max_	Conductance
HBS	HEPES-buffered saline
HEK	Human embryonic kidney
I/V	Current/voltage
I_Ba_	Barium current
I_CaL_	L-type calcium current
ISO	Isoproterenol
IVSd	Interventricular septum thickness during diastole
IVSs	Interventricular septum thickness during systole
k_act_	Slope of channel activation
KHB	Krebs–Henseleit buffer
L1482	Lysine 1482
LVIDd	Left ventricular internal diameter during diastole
LVIDs	Left ventricular internal diameter during systole
LVPWd	Left ventricular posterior wall thickness during diastole
LVPWs	Left ventricular posterior wall thickness during systole
MHC	α-myosin heavy chain
PKA	Protein kinase A
S1458	Serine 1458
S1487	Serine 1487
SEM	Standard error of the mean
THW/TL	Total heart weight to tibia length ratio
V_1/2_	Activation midpoint
wt	Wildtype
Y1481	Tyrosine 1481
