# Supplementary material for: A Seven Amino Acid Long Region in the Cardiac Cav1.2, Encompassing Ser1487, Is Essential for Mice Embryonic Development and “Fight or Flight” Responses
Source: Cells. 2026 Sep 14;15(18):1655. doi: 10.3390/cells15181655 (PMC13605837; doi:10.3390/cells15181655)
Supplement: Supplementary file 1 [file cells-15-01655-s001.zip › cells-4542636-supplementary.pdf]

Supplementary Materials for

A Seven Amino Acid Long Region in the Cardiac Cav1.2, Encompassing Ser1487, Is Essential for Mice Embryonic Development and "Fight or Flight" Responses

Catherine Jenkins & Henrietta Cserne Szappanos *et al.*

*Corresponding author. Email: livia.hool@uwa.edu.au

This PDF file includes:

Figures. S1 to S6
Tables S1 and S2

```
sp|Q01815|CAC1C_MOUSE                 ISFYMLCAFLIINLFVAVIMDNFDYLTRDWSILGPHHLDEFKRIWAEYDPEAKGRIKHLD   1516
sp|Q13936|CAC1C_HUMAN                 ISFYMLCAFLIINLFVAVIMDNFDYLTRDWSILGPHHLDEFKRIWAEYDPEAKGRIKHLD   1564
sp|P15381|CAC1C_RABIT                 ISFYMLCAFLIINLFVAVIMDNFDYLTRDWSILGPHHLDEFKRIWAEYDPEAKGRIKHLD   1546
sp|P22002|CAC1C_RAT                   ISFYMLCAFLIINLFVAVIMDNFDYLTRDWSILGPHHLDEFKRIWAEYDPEAKGRIKHLD   1545
tr|A0A8V0YQE9|A0A8V0YQE9_CHICK        ISFYMLCAFLIINLFVAVIMDNFDYLTRDWSILGPHHLDEFKRIWAEYDPEAKGRIKHLD   1521
tr|A0A8J1MJD2|A0A8J1MJD2_XENLA        ISFYMLCAFLIINLFVAVIMDNFDYLTRDWSILGPHHLDEFKRIWAEYDPEAKGRIKHLD   1511
tr|A0A8M9PU49|A0A8M9PU49_DANRE        VSFYMLCAFLIINLFVAVIMDNFDYLTRDWSILGPHHLDEFKRIWAEYDPEAKGRIKHLD   1540
tr|M9PDI1|M9PDI1_DROME                ISFYVLCSFLIINLFVAVIMDNFDYLTRDWSILGPHHLDEFIRLWSEYDPDAKGRIKHLD   2149
tr|Q8MQA1|Q8MQA1_CAEEL                ISFFMLCSFLVINLFVAVIMDNFDYLTRDWSILGPHHLEEFVRLWSEYDPDAKGRIKHLD   1405
                                      :**::**:**:***************************:** *:*:****:*********
```

Figure S1. Sequence alignment (Clustal Omega) of CACNA1C homologs/orthologs across different species, including the proximal C-terminal region of the channel. Highlighted are the 7 amino acids in this region which are highly conserved (including Ser1487 in mouse). On the left the name of the species and the protein sequence identifier are listed.

NFAT3 (140 kDa)

03_050321

mb#1

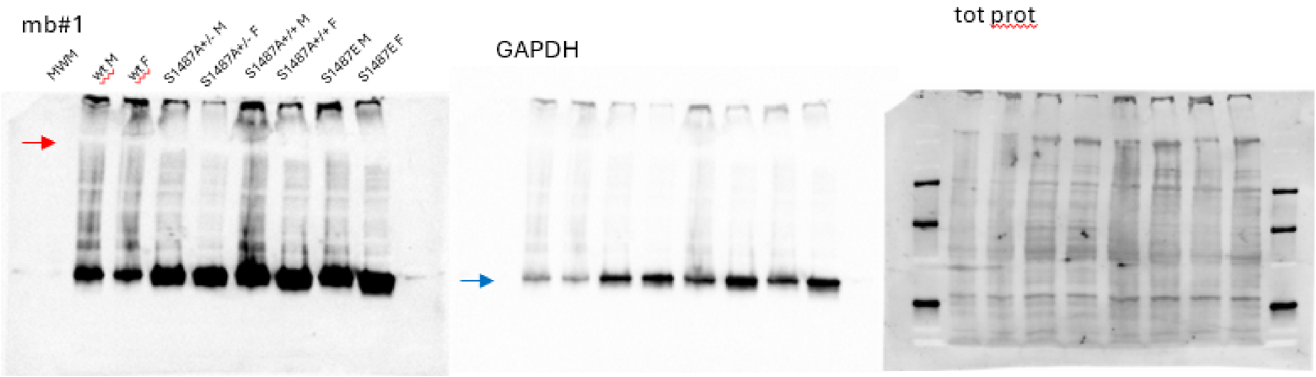

NFAT3 (140 kDa)

10_130521

mb#4

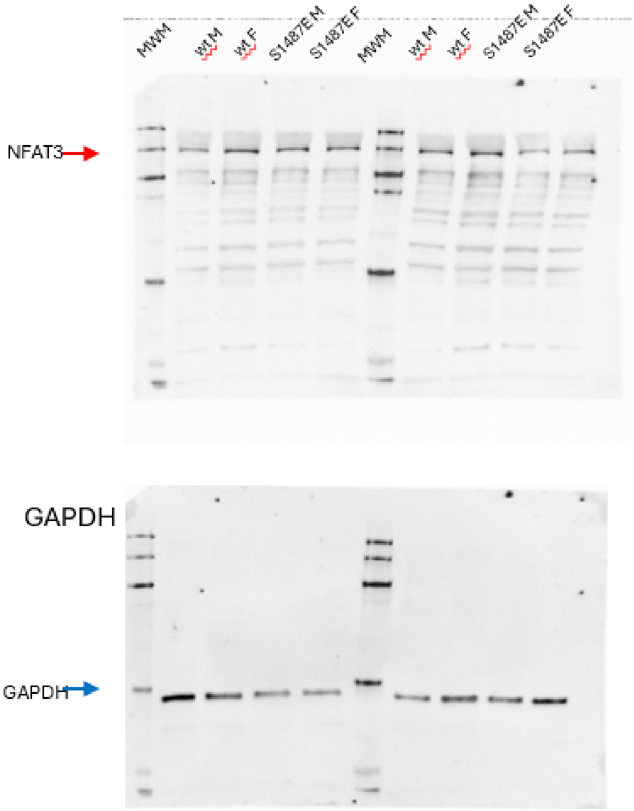

24_271022

mb#2

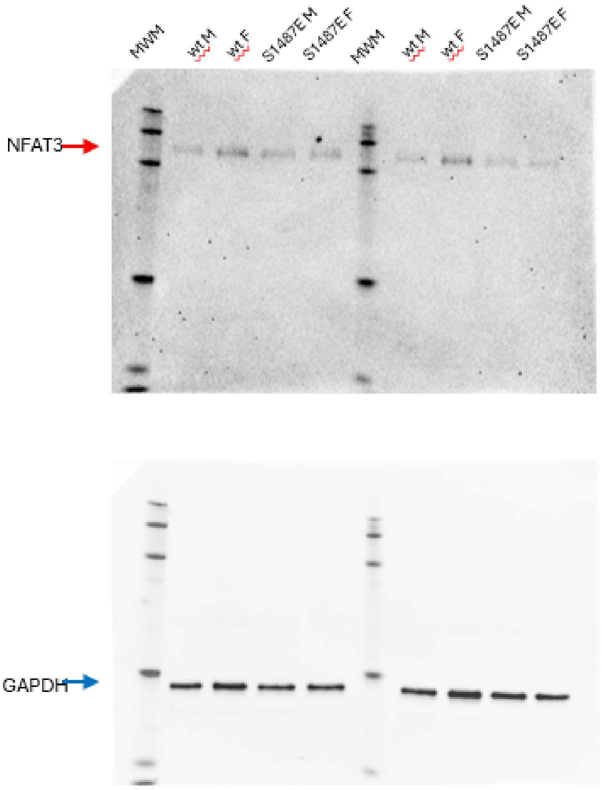

NFAT3 (140 kDa)

24_271022

mb#1

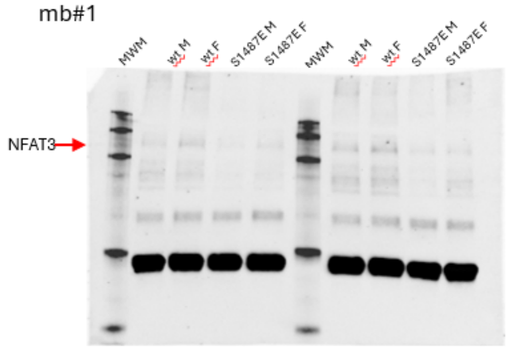

19_270721

mb#1

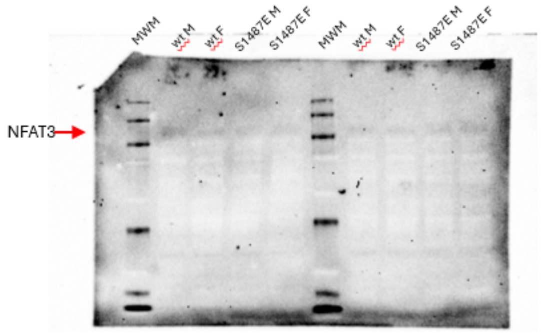

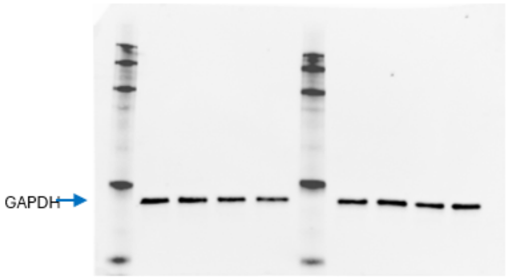

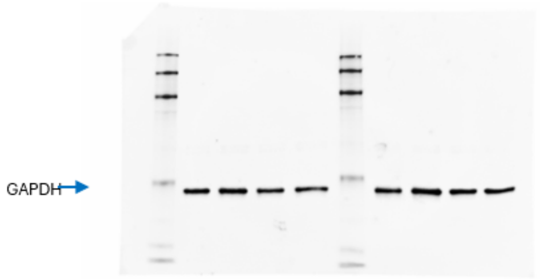

GATA4 (55 kDa)

19_270721

mb#3

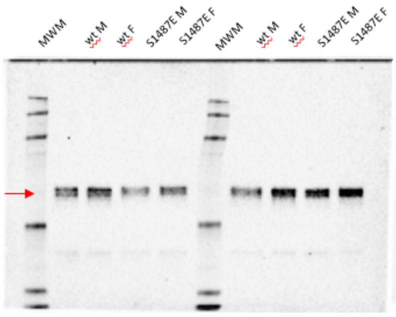

mb#4

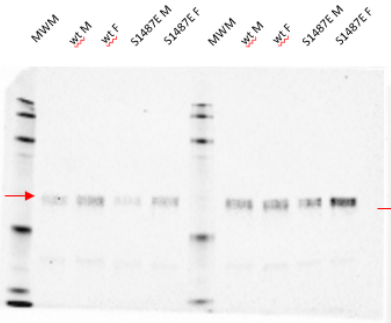

24_271022

mb#1

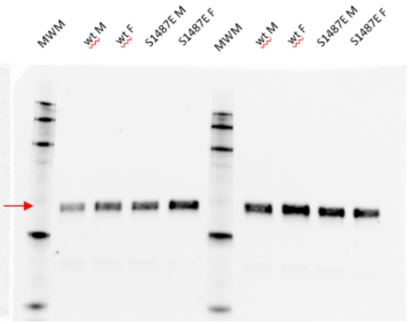

GAPDH

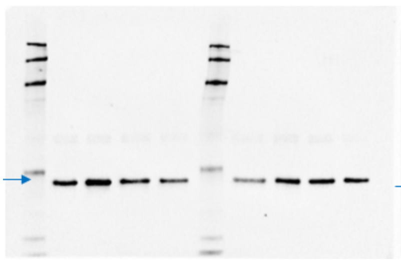

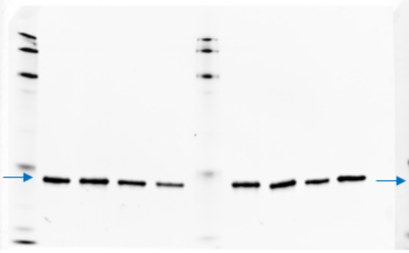

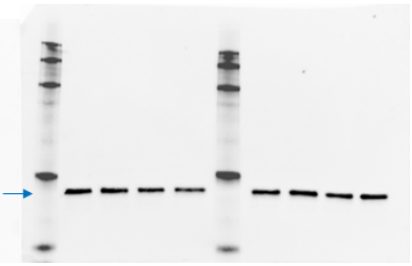

pGSK3β (46 kDa)

05_070521 mb#3 | 10_130521 mb#3 | 19_270721 mb#5

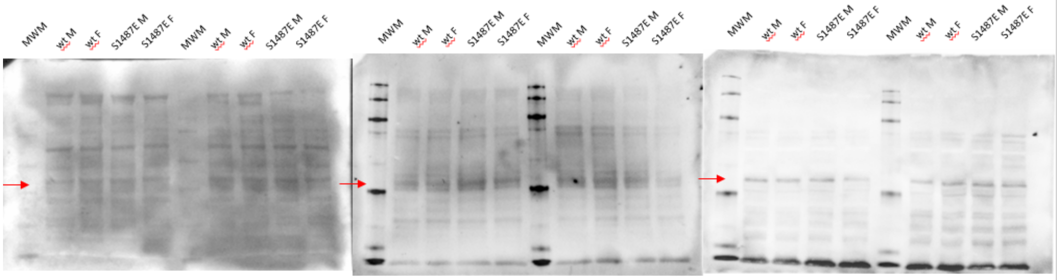

GAPDH

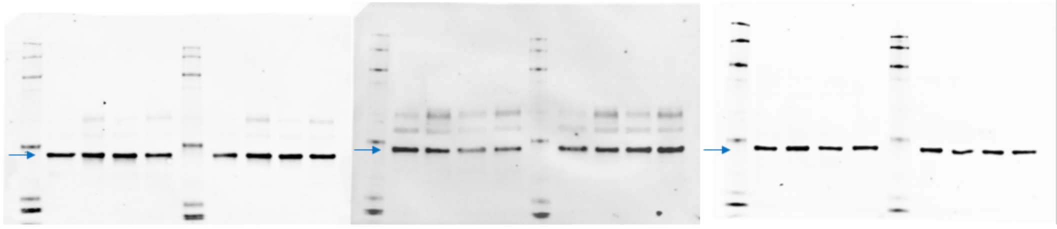

pGSK3αβ (51/46 kDa)

24_271022 mb#3 | 24_271022 mb#4

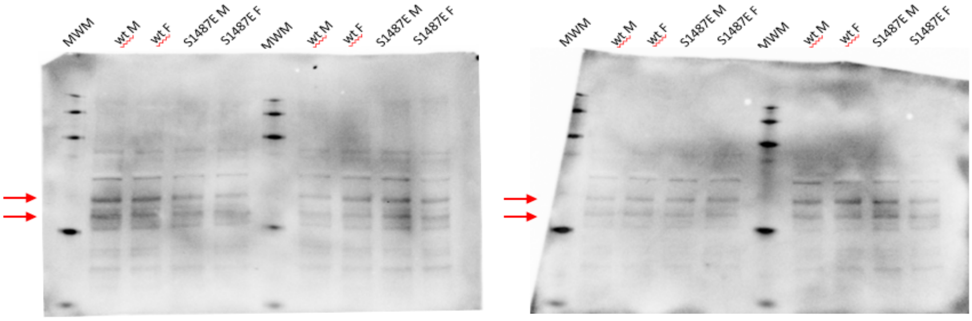

GAPDH

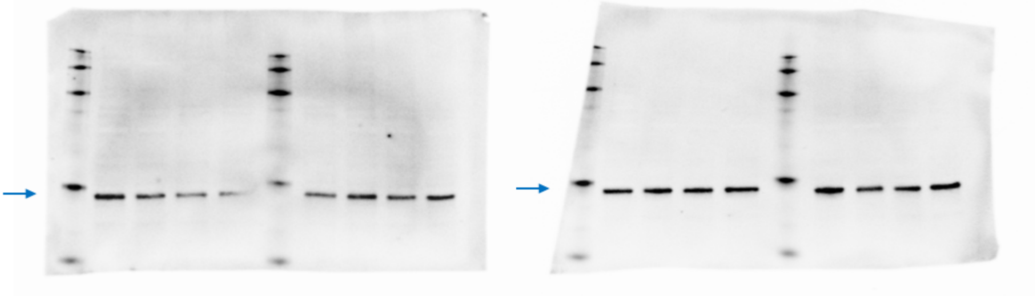

Figure S2. Full length Western blot images corresponding to cropped blot images shown in Figure 4A. Full length Western blots of NFAT3, GATA4, p-GSK3α, and p-GSK3β to investigate pathways involved in S1487E-mediated hypertrophy. Protein probed is normalized to the loading for GAPDH protein (MWM: molecular weight marker, mb: membrane, tot prot: total protein, M: male, F: female). Arrow indicates band corresponding to protein of interest.

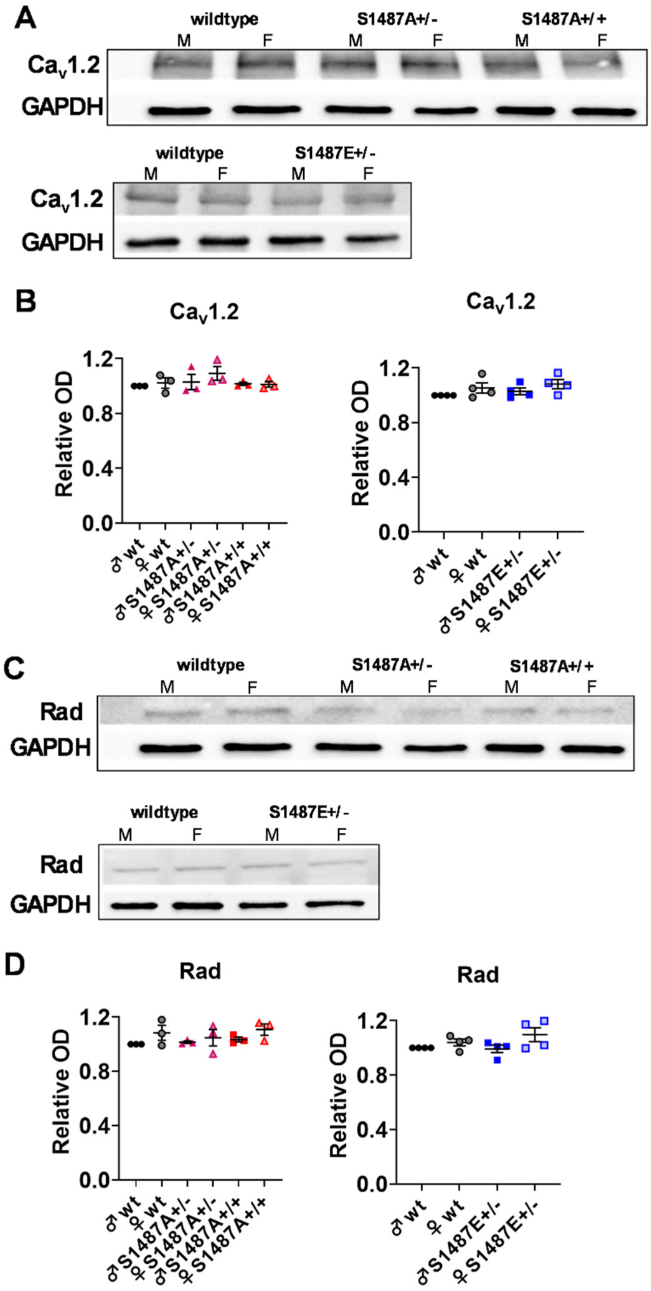

Figure S3. Western blot analysis of $Ca_V1.2$ and Rad expression There were no changes in expression of the $Ca_V1.2$ channel or Rad protein in S1487A and S1487E mutant mouse models. Protein probed is normalized to loading of GAPDH protein. Comparisons between genotypes performed using One Way ANOVA compared to wt male. Sex differences investigated by genotype matched gender comparisons using unpaired t-tests.

Cav1.2 (240kDa)

03_050321

mb#1

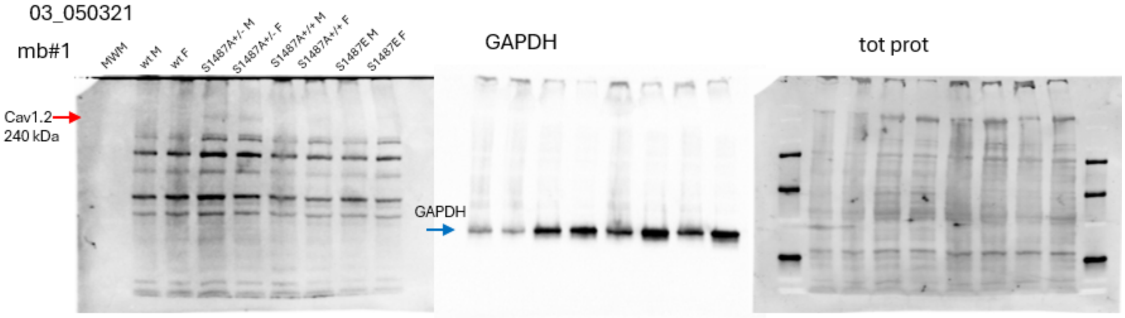

mb#2

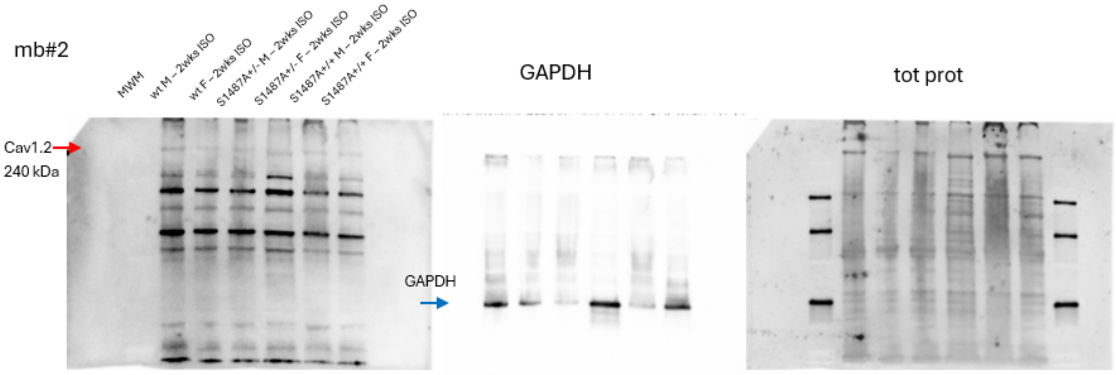

Cav1.2 (240kDa)

15_190321

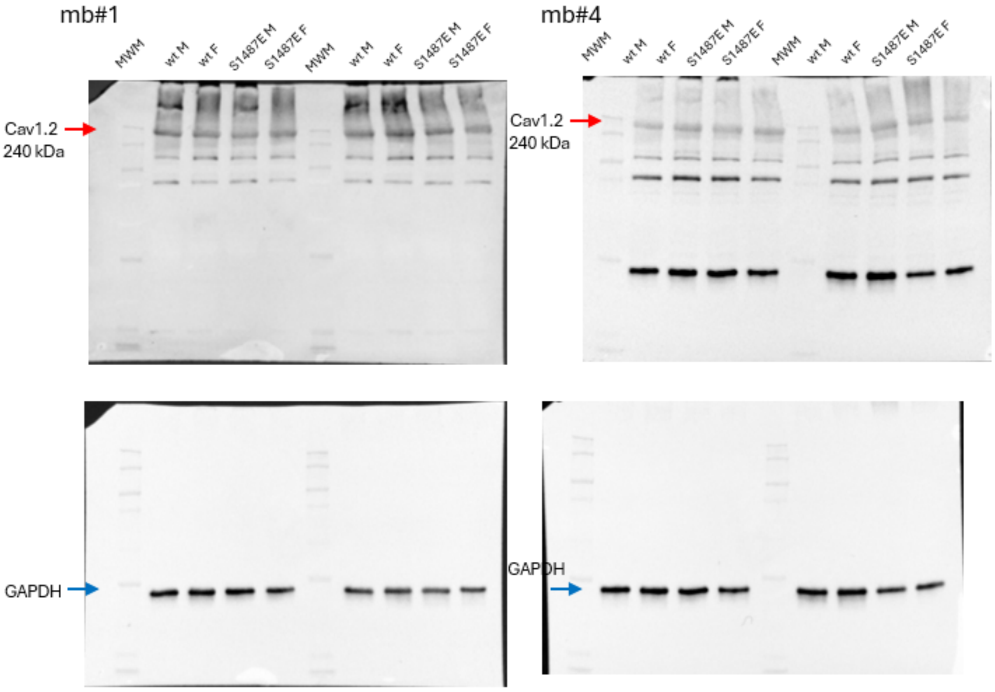

RRAD (37kDa)

03_050321

mb#1

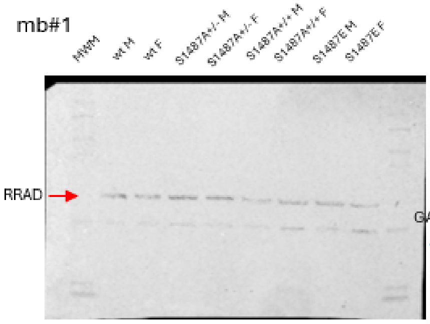

GAPDH

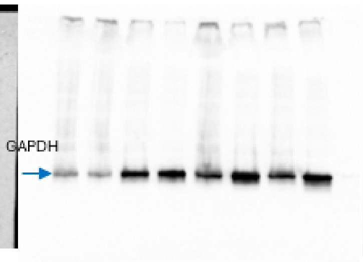

tot prot

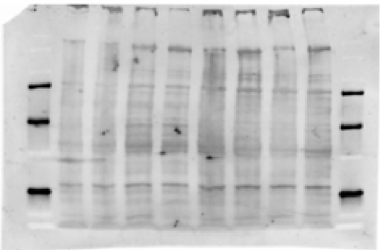

mb#2

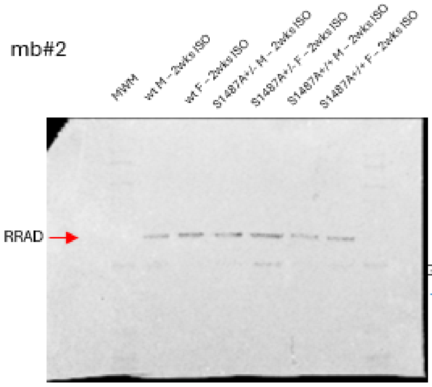

GAPDH

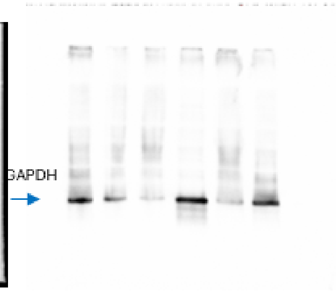

tot prot

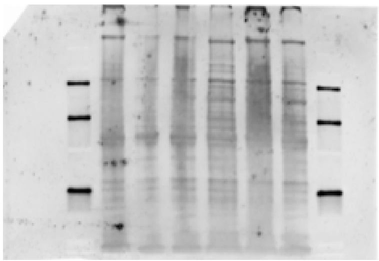

15_190321

mb#1 mb#2

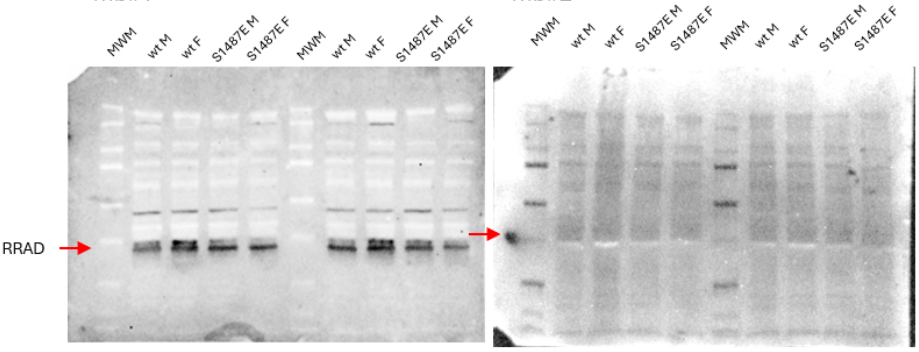

GAPDH

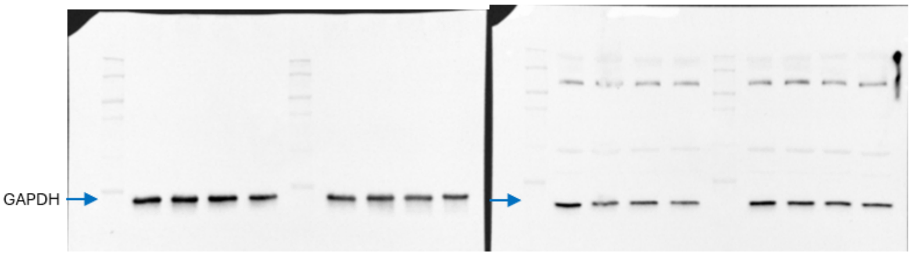

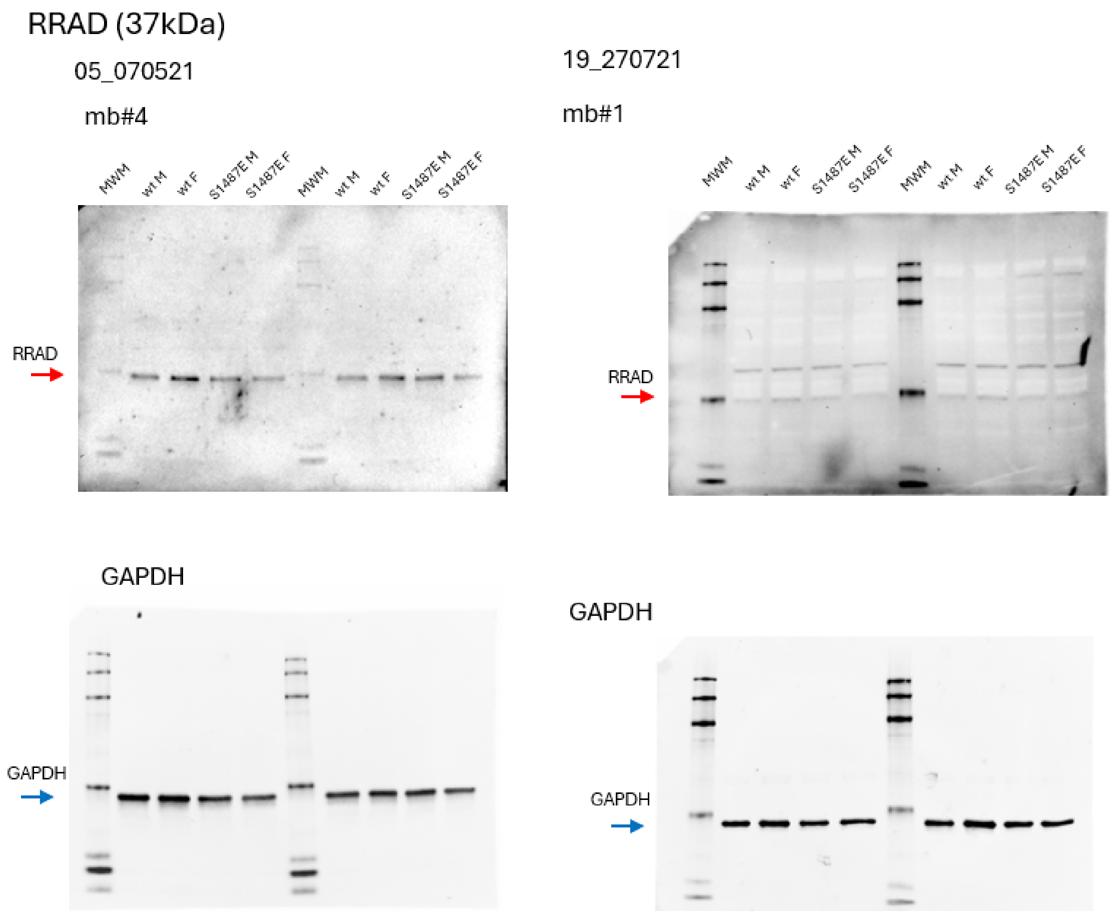

Figure S4. Full length Western blot images corresponding to cropped blot images shown in Supplementary Figure S3. Full length Western blots of Ca_v1.2 and Rad (RRAD) Protein normalized to loading of GAPDH protein. (MWM: molecular weight marker, mb: membrane, tot prot: total protein, M: male, F: female). Arrow indicates band corresponding to protein of interest.

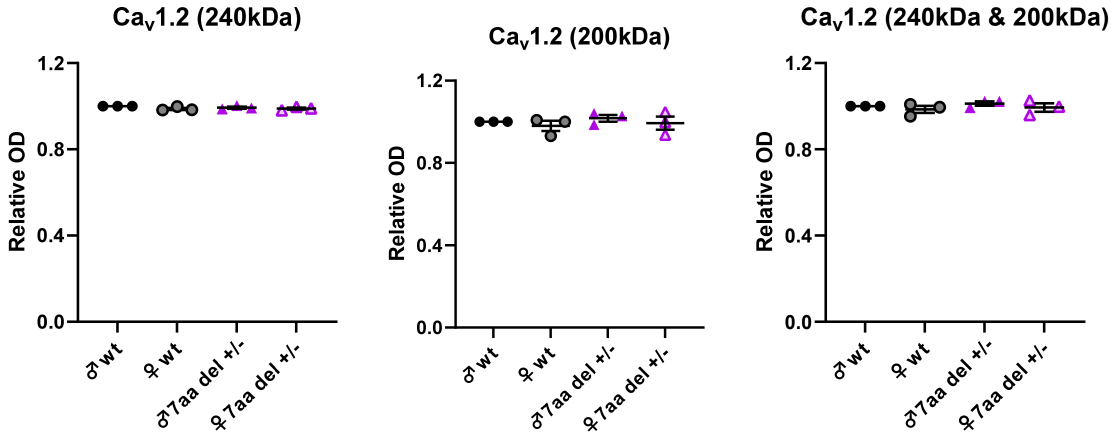

Figure S5. Western blot analysis of $Ca_V1.2$ expression There were no changes in expression of the $Ca_V1.2$ channel protein in the 7aa del$^{+/-}$ mutant mouse model. Comparisons between genotypes performed using One Way ANOVA compared to wt male. Sex differences investigated by genotype matched gender comparisons using unpaired t-tests.

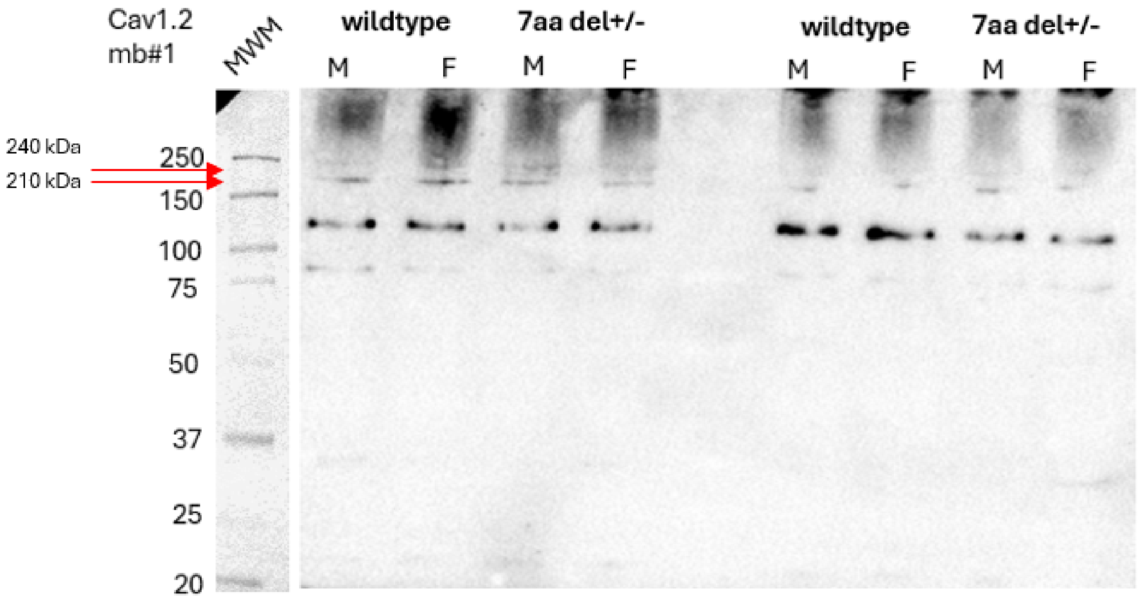
Cav1.2
mb#1
MWM
wildtype
7aa del+/-
wildtype
7aa del+/-
M
F
M
F
M
F
M
F
240 kDa
210 kDa
250
150
100
75
50
37
25
20

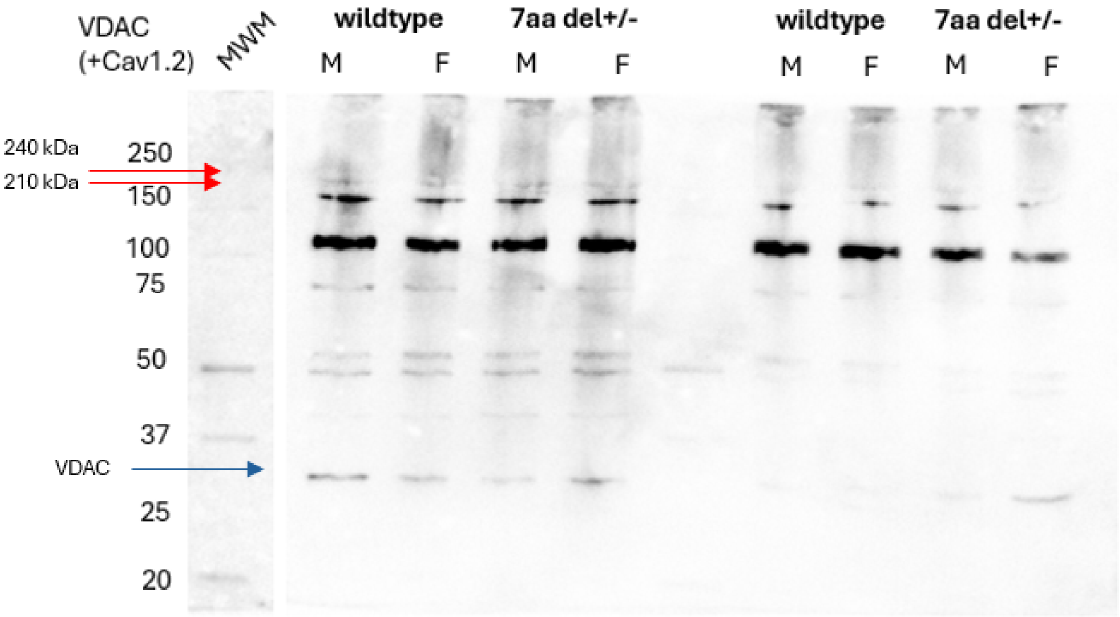
VDAC
(+Cav1.2)
MWM
wildtype
7aa del+/-
wildtype
7aa del+/-
M
F
M
F
M
F
M
F
240 kDa
210 kDa
250
150
100
75
50
37
VDAC
25
20

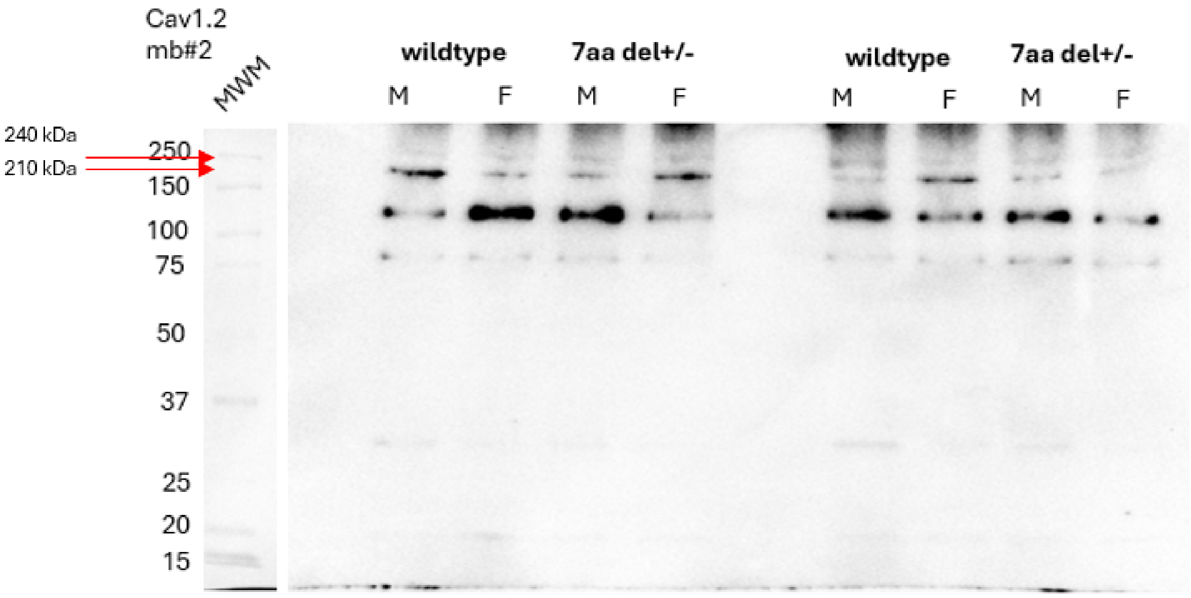
Cav1.2
mb#2
MWM
wildtype
7aa del+/-
wildtype
7aa del+/-
M
F
M
F
M
F
M
F
240 kDa
210 kDa
250
150
100
75
50
37
25
20
15

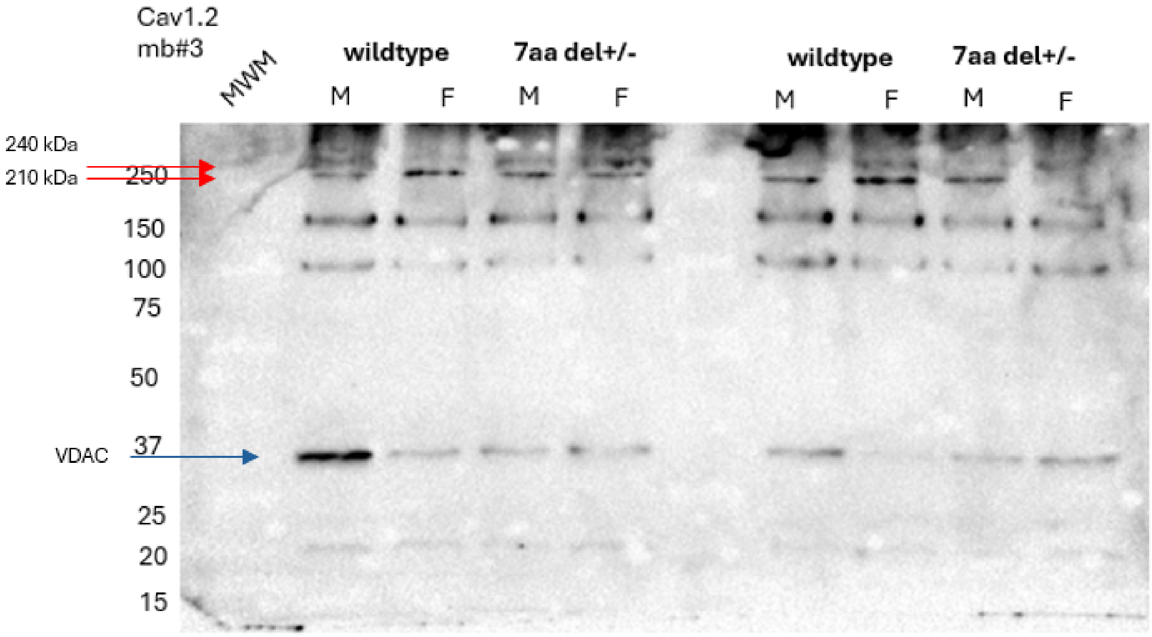
Cav1.2
mb#3
MWM
wildtype
7aa del+/-
wildtype
7aa del+/-
M
F
M
F
M
F
M
F
240 kDa
210 kDa
250
150
100
75
50
VDAC
37
25
20
15

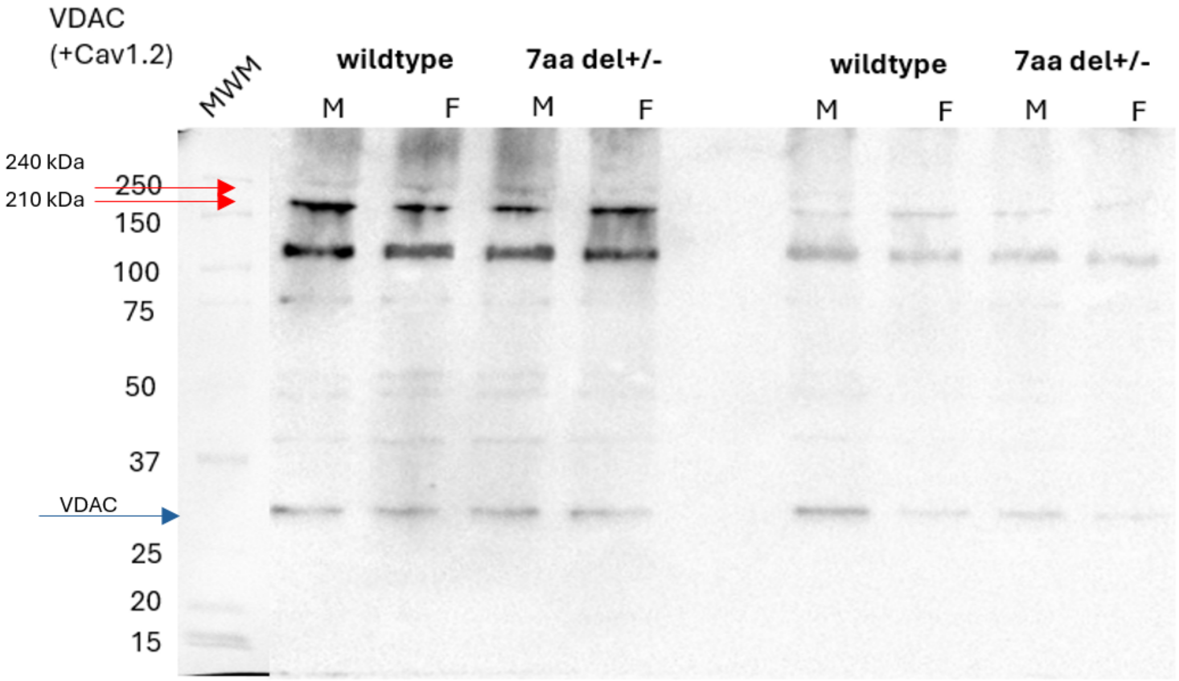

Figure S6. Full length Western blot images corresponding to cropped blot images shown in Supplementary Figure S5. Full length Western blots of $Ca_v1.2$. Protein normalized to loading of VDAC (MWM: molecular weight marker, mb: membrane, tot prot: total protein, M: male, F: female).

Table S1: Biophysical parameters recorded in adult cardiac myocytes at baseline and the fold change responses to acute β-adrenergic (AR) responses. Analyzed using One-Way ANOVA with Kruskal-Wallis test for multiple comparisons and Dunn's post-hoc test.

Baseline (prior to β-AR stimulation)	**wt (n=34)**	**S1487A +/+ (n=33)**		**S1487E +/- (n=31)**		**7aa del +/- (n=19)**	
	Mean ± SEM	*Mean ± SEM*	*p*	*Mean ± SEM*	*p*	*Mean ± SEM*	*p*
**Current Density (pA/pF)**	-8.37 ± 0.67	-10.59 ± 0.6	>0.9999[a], 0.578[b], 0.3681[c]	-12.12 ± 1	**0.0376**[a], **0.0032**[c]	-7.78 ± 0.43	>0.9999[a]
**Time-to-peak (ms)**	10.02 ± 0.55	7.49 ± 0.31	**0.0009**[a], **0.0005**[b], **0.0279**[c]	9.38 ± 0.47	>0.9999[a,c]	9.29 ± 0.46	>0.9999[a]
**Activation Tau (ms)**	3.05 ± 0.25	2.05 ± 0.11	>0.9999[a,c], **0.048**[b]	3.69 ± 0.3	0.3179[a], **0.0139**[c]	2.52 ± 0.24	0.9334[a]
**Activation A.U.C (pA.ms)**	6043.13 ± 351.1	6532.2 ± 332.8	0.9401[a], **<0.0001**[b], 0.9958[c]	10931 ± 1265.4	**<0.0001**[a], **0.0003**[c]	7007.94 ± 671.7	0.8901[a]
**Inactivation Tau 1 (ms)**	29.31 ± 3.08	28.10 ± 2.05	>0.9999[a,c], 0.3117[b]	24.46 ± 3.01	0.9441[a], >0.9999[c]	25.82 ± 2.76	>0.9999[a]
**Inactivation Tau 2 (ms)**	8.24 ± 0.95	9.04 ± 0.7	>0.9999[a,c], **0.0118**[b]	5.12 ± 0.88	0.1741[a], 0.1439[c]	8.95 ± 1.3	>0.9999[a]
**Inactivation A.U.C (pA.ms)**	23596.6 ± 2091.6	28010.7 ± 2208	>0.9999[a,c], **0.0197**[b]	40481.4 ± 3843.8	**0.0001**[a], >0.9999[c]	33314.8 ± 2606.4	0.0875[a]
**V_{rev} (mV)**	33.1 ± 1.02	37.6 ± 0.84	**0.0084**[a], 0.9863[b], 0.9647[c]	38.22 ± 1.35	**0.0265**[a], 0.8719[c]	38.11 ± 1.14	**0.0084**[a]
**$V_{1/2}$ (mV)**	-23.04 ± 1.25	-26.03 ± 0.87	0.5907[a], >0.9999[b], **0.0088**[c]	-24.54 ± 1.57	>0.9999[a], **0.0231**[c]	-20.7 ± 0.98	0.4250[a]
**G_{max} (pS/pF)**	190.7 ± 11.3	206.5 ± 8.7	>0.9999[a,b], 0.6715[c]	224.72 ± 14.2	>0.9999[a,c]	168.34 ± 7.71	>0.9999[a]
**k_{act}**	3.52 ± 0.18	4.05 ± 0.2	0.4451[a], **0.0016**[b], >0.9999[c]	3.36 ± 0.36	0.3155[a], 0.1127[c]	4.08 ± 0.42	>0.9999[a]
Fold-change in response to β-AR stimulation	**wt (n=34)**	**S1487A +/+ (n=33)**		**S1487E +/- (n=31)**		**7aa del +/- (n=19)**	
	Mean ± SEM	*Mean ± SEM*	*p*	*Mean ± SEM*	*p*	*Mean ± SEM*	*p*
**Current Density (pA/pF)**	1.6 ± 0.09	1.1 ± 0.02	**<0.0001**[a], >0.9999[b], 0.4312[c]	1.1 ± 0.05	**<0.0001**[a], 0.1960[c]	1.3 ± 0.06	**0.0054**[a]
**Time-to-peak (ms)**	0.8 ± 0.03	0.93 ± 0.03	0.6621[a], 0.4561[b], >0.9999[c]	1.0 ± 0.06	0.0809[a], 0.2533[c]	1.0 ± 0.03	**0.0247**[a]
**Activation Tau (ms)**	1.02 ± 0.09	0.88 ± 0.05	>0.9999[a,b], **0.0126**[c]	1.1 ± 0.13	>0.9999[a], 0.3399[c]	1.3 ± 0.12	0.1362[a]
**Activation A.U.C (pA.ms)**	1.5 ± 0.05	1.1 ± 0.03	**<0.0001**[a], >0.9999[b], 0.1532[c]	1.5 ± 0.3	**0.0003**[a], 0.3593[c]	1.3 ± 0.06	0.6276[a]
**Inactivation Tau 1 (ms)**	1.45 ± 0.16	1.3 ± 0.12	>0.9999[a,b,c]	1.1 ± 0.13	>0.9999[a,c]	1.2 ± 0.12	>0.9999[a]
**Inactivation Tau 2 (ms)**	1.1 ± 0.09	0.98 ± 0.07	0.5083[a], 0.1544[b], >0.9999[c]	2.0 ± 0.38	>0.9999[a], 0.1338[c]	1.6 ± 0.56	0.4002[a]
**Inactivation A.U.C (pA.ms)**	2.6 ± 0.18	1.85 ± 0.1	**0.0148**[a], 0.3152[b], >0.9999[c]	1.7 ± 0.2	**<0.0001**[a], >0.9999[c]	1.8 ± 0.11	**0.0206**[a]
**V_{rev} (mV)**	1.2 ± 0.04	1.0 ± 0.02	**0.0045**[a], >0.9999[b,c]	1.0 ± 0.06	0.0695[a], >0.9999[c]	1.0 ± 0.03	0.0515[a]
**$V_{1/2}$ (mV)**	1.3 ± 0.05	1.1 ± 0.03	>0.9999[a,b,c]	1.2 ± 0.08	0.4297[a], >0.9999[c]	1.2 ± 0.03	>0.9999[a]
**G_{max} (pS/pF)**	1.2 ± 0.05	1.0 ± 0.02	0.2124[a], >0.9999[b,c]	1.0 ± 0.05	**0.0176**[a], >0.9999[c]	1.1 ± 0.05	0.4478[a]
**k_{act}**	0.93 ± 0.04	0.98 ± 0.03	0.8903[a], 0.0737[b], 0.7539[c]	1.2 ± 0.12	**0.0243**[a], **0.0189**[c]	0.9 ± 0.06	0.9973[a]

a: vs wt, b: vs S1487E +/-, c: vs 21bp del +/-, n = number of cells

Table S2: Biophysical parameters assessed in HEK cells transfected with wt-Ca_v1.2. Data presented as Mean ± SEM

	**wt-Ca_v1.2 (no Rad) (n=13)** *Baseline*
**Ipeak (pA)**	-1866.5 ± 194.2
**I (pA/pF)**	-88.1 ± 7.9
**ttp (ms)**	14.6 ± 1.5
**$V_{1/2}$ (mV)**	-12.0 ± 1.4
**k_{act} (mV)**	2.7 ± 0.2
**G_{max} (pS/pF)**	68.1 ± 13.6
**τ_{act} (ms)**	2.4 ± 0.4
**AUC_{act} (pA*ms)**	16410.7 ± 1668
